# Metformin is a metabolic modulator and radiosensitiser in rectal cancer

**DOI:** 10.3389/fonc.2023.1216911

**Published:** 2023-08-03

**Authors:** Croí E. Buckley, Rebecca M. O’Brien, Timothy S. Nugent, Noel E. Donlon, Fiona O’Connell, John V. Reynolds, Adnan Hafeez, Diarmuid S. O’Ríordáin, Robert A. Hannon, Paul Neary, Reza Kalbassi, Brian J. Mehigan, Paul H. McCormick, Cara Dunne, Michael E. Kelly, John O. Larkin, Jacintha O’Sullivan, Niamh Lynam-Lennon

**Affiliations:** ^1^ Department of Surgery, School of Medicine, Trinity Translational Medicine Institute, Trinity College Dublin, Dublin, Ireland; ^2^ Trinity St. James’s Cancer Institute, St. James’s Hospital, Trinity College Dublin, Dublin, Ireland; ^3^ Department of Surgery, Beacon Hospital, Dublin, Ireland; ^4^ Gastrointestinal Medicine and Surgery (GEMS) Directorate, St. James’s Hospital, Dublin, Ireland

**Keywords:** metformin, rectal cancer, colorectal, radiosensitiser, energy metabolism, radioresistance

## Abstract

Resistance to neoadjuvant chemoradiation therapy, is a major challenge in the management of rectal cancer. Increasing evidence supports a role for altered energy metabolism in the resistance of tumours to anti-cancer therapy, suggesting that targeting tumour metabolism may have potential as a novel therapeutic strategy to boost treatment response. In this study, the impact of metformin on the radiosensitivity of colorectal cancer cells, and the potential mechanisms of action of metformin-mediated radiosensitisation were investigated. Metformin treatment was demonstrated to significantly radiosensitise both radiosensitive and radioresistant colorectal cancer cells *in vitro*. Transcriptomic and functional analysis demonstrated metformin-mediated alterations to energy metabolism, mitochondrial function, cell cycle distribution and progression, cell death and antioxidant levels in colorectal cancer cells. Using *ex vivo* models, metformin treatment significantly inhibited oxidative phosphorylation and glycolysis in treatment naïve rectal cancer biopsies, without affecting the real-time metabolic profile of non-cancer rectal tissue. Importantly, metformin treatment differentially altered the protein secretome of rectal cancer tissue when compared to non-cancer rectal tissue. Together these data highlight the potential utility of metformin as an anti-metabolic radiosensitiser in rectal cancer.

## 1 Introduction

Colorectal cancer (CRC) has the 3^rd^ highest incidence rate of cancer worldwide, and accounts for the 2^nd^ highest cancer mortality rate (1). An estimated one in three cases of CRC occur in the lower bowel, or the rectum, the incidence of which are predicted to rise exponentially in the coming years (2). Alarmingly, this predicted rise in the incidence of rectal cancer is thought to be largely driven by an increase in rectal cancer incidence in young adults (<50 years) (3–6), who often present with advanced stage disease, more aggressive histopathologic characteristics and poorer prognoses (7, 8).

The current standard of care for locally-advanced rectal cancer is neoadjuvant chemoradiation therapy (neoCRT), followed by surgical resection. Patients typically receive long-course radiation therapy, combined with 5-fluorouracil (5-FU) based chemotherapy prior to total mesorectal excision (TME) (9). Increasingly, patients are being considered for total neoadjuvant therapy. TME is a major procedure and is associated with increased risk of perioperative mortality and morbidity and has long term impacts on the quality of life for cancer survivors (10). Therefore, there is an increasing interest in the identification of treatment strategies that can facilitate organ preservation (11). The attainment of a pathological complete response (pCR) following neoCRT, which is characterised by no viable tumour cells, is associated with low recurrence rates and improved survival outcomes (12), highlighting the potential for organ preservation in these patients. Unfortunately, only an estimated 20-30% of patients achieve a pCR following neoCRT (13–16). Consequently, there is an unmet need to identify novel treatment strategies to boost the response to neoCRT in rectal cancer patients to facilitate organ preservation and improve outcomes for patients.

Radiosensitising drugs aim to enhance radiation-induced damage in cancer cells, while sparing surrounding healthy tissue. To date, few radiosensitising drugs have been approved for use clinically (17). Increasing evidence supports a role for altered energy metabolism in the radioresistance of cancer (18–20). We have previously demonstrated that metabolic reprogramming is associated with a radioresistant phenotype in both *in vitro* and *ex vivo* models of oesophageal adenocarcinoma (21, 22) and rectal adenocarcinoma (23).

Metformin is a clinically approved drug used for the management of type II diabetes (24), and has been demonstrated to act as an inhibitor of oxidative phosphorylation, through inhibition of complex I of the Electron Transport Chain (ETC) (25, 26). Observational studies have demonstrated that diabetics with rectal or oesophageal cancer, treated with metformin during cancer treatment, display enhanced responses to cancer therapy (27–29), indicating the potential utility of metformin as a radiosensitising drug. Previous research has demonstrated the efficacy of metformin as a radiosensitiser in models of colon cancer (30, 31). However, the potential of metformin as a novel anti-metabolic radiosensitiser in rectal cancer is largely unknown.

This study demonstrates, through real-time live cell metabolic profiling and transcriptomic profiling, that metformin significantly alters energy metabolism in both *in vitro* and *ex vivo* models of rectal adenocarcinoma and importantly, radiosensitises radioresistant rectal cancer cells to clinically-relevant doses of X-ray radiation. Furthermore, we demonstrate that metformin-induced radiosensitisation may be mediated in part through alterations to cell cycle, cell death and oxidative stress. This study also importantly demonstrates that metformin treatment significantly alters the metabolic profile and inflammatory secretome in rectal cancer biopsies, with minimal impact on non-cancer rectal tissue, supporting its potential as a radiosensitising agent in rectal cancer.

## 2 Materials and methods

### 2.1 CRC adenocarcinoma cell lines

SW837 rectal cancer and HCT116 colon cancer cell lines were obtained from the European Collection of Cell Culture (ECACC). SW837 cells were maintained in Leibovitz’s (L-15) culture media (Lonza, Basel, Switzerland), supplemented with penicillin-streptomycin (1%) (v/v) (Lonza), foetal bovine serum (FBS) (10%) (v/v) (Gibco, Waltham, MA, USA), and L-Glutamine (Lonza) (1%) (v/v) (complete medium), in non-vented flasks. HCT116 colon cancer cells were maintained in Roswell Park Memorial Institute (RPMI)-1460 medium (Gibco), supplemented with penicillin-streptomycin (1%) (v/v), and FBS (10%) (v/v) (complete medium) (complete RPMI (cRPMI)), in vented flasks. Cell lines were cultured at 37°C, in 5% CO_2_/95% humidified air.

### 2.2 Crystal violet assay

SW837 cells were fixed via the addition of 50 µL 1% glutaraldehyde [(v/v) in phosphate buffered saline (PBS)], per well) for 15 min, at room temperature (RT°). HCT116 cells were fixed with cold 4% paraformaldehyde (PFA) (v/v) for 10 min, at RT°. The fixative was removed and cells were washed with 50 µL PBS. Cells were stained with 0.1% crystal violet (w/v) (in dH_2_O) for 30 min at RT°. Cells were gently washed with 50 µL water and allowed to air dry overnight at RT°. The crystal violet dye was then eluted via the addition of 50 µL 1% Triton-X100 (in PBS) on an orbital shaker for ~ 1 h, or until the dye fully eluted, at RT°. The absorbance was read at 595 nm on a VersaMax microplate reader (Molecular Devices, Sunnyvale, CA USA).

### 2.3 Real-time metabolic profiling of cell lines

Cells were seeded at optimised seeding densities (HCT116: 10,000 cells/well, SW837: 30,000 cells/well in a final volume of 100 µL cRPMI) in a 24-well cell culture XFe24 microplate (Agilent Technologies, Santa Clara, CA, USA) and allowed to adhere at 37°C, 5% CO_2_/95% humidified air. At 5 h post seeding, an additional 150 µL complete media was added to each well. Following 24 h, medium was removed to waste, and cells were treated with metformin (2.5 or 10 mM) (Sigma Aldrich, St. Louis, MO, USA) or vehicle control (H_2_O) diluted in cRPMI. Following 24 h, treatment was removed and cells were washed with unbuffered Seahorse XF Base DMEM (Agilent*)* (supplemented with 10 mM glucose (Sigma), 10 mM sodium pyruvate (Sigma) and L-glutamine) and placed in a non-CO_2_ incubator for 1 h at 37°C. OCR and ECAR were measured using the Seahorse XFe24 Extracellular Flux Analyser (Agilent). Three baseline measurements of OCR and ECAR were taken over 24 min, consisting of 2 repetitions of mix (3 min)/wait (2 min)/measurement (3 min), to establish basal respiration. All OCR/ECAR readings were normalised using the crystal violet assay.

### 2.4 Mitochondrial function

Three surrogate markers of mitochondrial function, reactive oxygen species (ROS), mitochondrial membrane potential and mitochondrial mass were assessed using a series of fluorescent probes (2,7 DCF), Rhodamine-123 and MitoTracker Green^FM^), as previously described (21). Briefly, HCT116 cells (10,000 cells per well) and SW837 cells (30,000 cells per well) were seeded in triplicate in a 96-well plate and placed at 37°C in 5% CO_2_/95% humidified air. At 24 h post seeding, the medium was removed and cells were treated with metformin (2.5 or 10 mM) or vehicle control (H_2_O) diluted in cRPMI. At 24 h post treatment, the media was removed and cells were incubated with a fluorescent probe {in PBS with magnesium [PBS (Mg^2+^)]} for 30 min, in the dark at 37°C. The probe was removed, fresh PBS was added and the fluorescence was immediately read using a FLx800 Fluorescence microplate reader (Mason Technology). Fluorescence values were subsequently normalised to cell number using the crystal violet assay.

### 2.5 X-ray radiation

All irradiations were performed using an X-Strahl cabinet X-ray irradiator (RS225) (X-Strahl) at a dose rate of 1.74 Gy/min.

### 2.6 Clonogenic assay

HCT116 and SW837 cells in the exponential growth phase were harvested, seeded in cRPMI into 6-well plates at optimised cell densities (HCT116: 0 Gy 500 cell/well, 1.8 Gy 1000 cells/well. SW837: 0 Gy 3000 cells/well, 1.8 Gy 5000 cells/well) and were allowed to adhere at 37°C in 5% CO_2_/95% humidified air overnight. Following 24 h incubation, medium was removed to waste, and cells were treated with metformin (1, 2.5 or 10 mM) or vehicle control (H_2_O) diluted in cRPMI. After 24 h, cells were exposed to X-ray radiation at 1.8 Gy or were mock-irradiated. For 5-FU treatments, cells were treated with 5-FU (15 µM) or DMSO control (0.001%) for 6 h prior to irradiation. At 24 h post-irradiation, treatment medium was removed and replenished with 1.5 mL/well cRPMI. Cells were incubated at 37°C in 5% CO_2_/95% humidified air for 7-14 days, until colonies formed but did not merge.

### 2.7 Fixation, staining and counting of clonogenic assay

For SW837 cells, a volume of 500 µL fixing/staining solution (0.05% (w/v) crystal violet (Sigma), 25% (v/v) Methanol (Sigma) in PBS) was added to each well and incubated for 30 min, at RT°. For HCT116 cells, a volume of 500 µL 4% PFA (4°C) (Santa Cruz Biotechnology, Inc., Dallas, TX, USA) was added to the well and incubated for 10 min at RT°. Colonies were stained with crystal violet solution (0.05% (w/v)) for 30 min at RT°. The stain was removed to waste, and the wells gently washed with water. Plates were then left to air-dry overnight. Colonies were counted using a PC-software operated colony counter (Gelcount™, Oxford Optronix Ltd., Abingdon, UK, version 1.2.1). Plating efficiencies (PEs), based on control colony counts, were calculated using the following formula: PE = no. colonies/no. cells seeded. The surviving fraction (SF) was calculated using the following formula: SF = No. colonies formed after treatment/(No. cells seeded x PE) as previously described (32).

### 2.8 Assessment of cell cycle

Cells were seeded into 12-well plates at optimised densities (HCT116: 150,000 cells/well, SW837: 200,000 cells/well) in cRPMI and allowed to adhere overnight at 37°C in 5% CO_2_/95% humidified air. At 24 h, medium was removed and cells were treated with metformin (10 mM) or vehicle control (H_2_O) diluted in cRPMI. Twenty-four hours later, cells were mock-irradiated or exposed to 1.8 Gy X-ray radiation. At, 6 h, 10 h, or 24 h post-irradiation, cells were collected into 5 mL flow tubes, and stained with propidium iodide (PI). Samples were acquired, with a minimum of 10,000 events collected, excluding doublets, using the FACSCanto II flow cytometer (BD Biosciences). PI was measured on the PerCP-Cy5 channel. Data were analysed using FlowJo™ Version 10.6.2.

### 2.9 Assessment of apoptosis by flow cytometry

Cells were seeded in cRPMI into 12-well plates at optimised seeding densities (HCT116: 400,000 cells/well, SW837: 500,000 cells/well) and allowed to adhere overnight at 37°C in 5% CO_2_/95% humidified air. At 24 h, medium was removed and cells were treated with metformin (10 mM) or vehicle control (H_2_O) diluted in cRPMI. After 24 h, cells were irradiated with 1.8 Gy X-ray radiation, while controls were mock-irradiated. At 24 h post radiation, supernatants and cells were collected into 5 mL flow tubes. Cells were stained with Annexin-V-FITC for 15 min at 4°C. Cells were then stained with PI prior to acquisition of 40,000 cells per tube, excluding doublets using the FACSCanto II flow cytometer. Annexin-V-FITC was measured on the FITC channel, while PI was measured on the PerCP-Cy5 channel. Data were analysed using FlowJo™ Version 10.6.2.

### 2.10 Glutathione GSH/GSSG-Glo™ assay

Cells were seeded in cRPMI in white-walled 96-well plates (Promega, Madison, WI, USA) at a density of 15,000 cells/well. At 24 h post seeding, medium was removed to waste, and cells were treated with 100 µL of metformin (10 mM) or vehicle control (H_2_O) diluted in cRPMI and incubated at 37°C in 5% CO_2_/95% humidified air. After 24 h treatment, media was removed to waste and GSH/GSSG levels measured using the GSH/GSSG-Glo™ luminescent assay (Promega), according to the manufacturer’s instructions. Luminescence was measured at 1 s integration time using the Explorer Luminometer (Promega). An identical plate of cells was set up and treated as with the experimental plate and was used for normalisation of assay results by crystal violet assay.

### 2.11 RNA isolation and quantification

SW837 cells were treated with metformin (10 mM) or vehicle control (H_2_O) diluted in cRPMI for 24 h. Total RNA was isolated from cells using the miRNeasy® Mini Kit (Qiagen, Hilden, Germany), according to manufacturer instructions. RNA was quantified using a Nanodrop 1000 spectrophotometer version 3.1 (Thermo Fisher Scientific, Dublin, Ireland).

### 2.12 Transcriptomic profiling

Transcriptomic profiling was conducted utilising mRNA sequencing using the Lexogen QuantSeq 3’ mRNA-Seq. RNA samples were prepared for sequencing using the QuantSeq™ 3’ mRNA-Seq Library prep kit (Lexogen, Vienna, Austria), according to the manufacturer instructions, using a starting volume of 50 ng RNA. An equal molar amount of the purified library was pooled for sequencing, with a loading concentration of 320 pM loaded onto the NovaSeq flowcell. Sequencing was performed using the NovaSeq 6000 (Illumina, San Diego, CA, USA), and an SP v1.5 sequencing kit (Illumina) with 1 x 100bp reads, as per manufacturer instructions.

### 2.13 Transcriptomic data analysis

Raw files were assessed using the BlueBee™ Bioinformatics platform (Lexogen). Raw reads were trimmed and aligned for automated gene counting. Once gene reads and counts were complete, differential expression analysis was performed using the DESeq2 R script extension within BlueBee software.

### 2.14 IPA analysis

Significantly differentially expressed genes, and corresponding Log_2_ Fold Change values were imported to IPA bioinformatics software (Qiagen, Redwood City, CA, USA, Winter Release 2021). Core analysis in IPA was performed, which utilises the Qiagen Knowledge Base, to identify networks and predict specific biological function and pathway involvement in the uploaded experimental transcriptomic dataset. Canonical Pathway Analysis in IPA, utilising the Qiagen Knowledge Base, was utilised to predict involvement and activation or inhibition of specific biological pathways in the experimental dataset. The *p*-value denotes the significance between the overlap of input experimental data and the Ingenuity Knowledge Base, indicating confidence in pathway involvement. The Z-score refers to software prediction of the activation or inhibition of each affected canonical pathway, with a Z-score ≥ 2, or ≤ -2 indicating significant activation or inhibition of each pathway, respectively.

### 2.15 Patient recruitment and ethics

Ethical approval for patient sample collection for this study was granted by the Joint St. James’s Hospital/AMNCH ethical review board (Reference 10/11/2011) and the Beacon Hospital Research Ethics Committee (Reference BEA0139). Patients undergoing lower gastrointestinal investigations or endoscopy for rectal cancer diagnosis were recruited between October 2020 and January 2022 from St. James’s Hospital, Dublin and Beacon Hospital, Dublin. Pre-treatment rectal tumour biopsies were obtained from consenting patients at diagnostic endoscopy. Normal (non-cancer) rectal tissue biopsies were obtained during colonoscopy from consenting patients who did not have a cancer diagnosis. Histological confirmation of tumour tissue and non-malignant tissue in biopsies was performed by an experienced pathologist using haematoxylin and eosin staining.

### 2.16 Protein isolation and quantification from biopsies

Protein was isolated from patient tissue biopsies using an AllPrep DNA/RNA/Protein Mini Kit (Qiagen), according to manufacturers’ instructions. To quantify the protein content of patient biopsies, the Pierce bicinchoninic acid (BCA) protein assay kit (Thermo Fisher Scientific) was utilised, according to the manufacturer’s instructions.

### 2.17 Real-time metabolic profiling of rectal tumour and non-cancer rectal tissue biopsies

Two biopsies per patient were collected at colonoscopy, placed into an individual well of an XF24 Islet Capture Microplate (Agilent Technologies) and secured into place by islet capture screens. A volume of 1 mL complete M199 (Gibco) [supplemented with FBS (10%), penicillin-streptomycin (1%), Fungizone™ (1%), gentamycin (0.1%) and insulin (1 µg/mL)] was placed in each well. The plate was placed at 37°C, in 5% CO_2_/95% humidified air for 30 min to equilibrate. Three basal measurements of OCR and ECAR were measured over 24 min of three repeats of mix (3 min)/wait (2 min)/measurement (3 min) using the Seahorse XFe24 analyser. The biopsies were treated with metformin (10 mM) or H_2_O control in complete M199 medium (1 mL volume). Biopsies were cultured for 24 h at 37°C in 5% CO_2_/95% humidified air, and basal measurements of OCR and ECAR measured over 24 min as described. Biopsies and matching tumour conditioned media (TCM) or non-cancer conditioned media (NCM) were collected, snap-frozen in liquid nitrogen, and stored at -80°C until required. The effect of treatment was assessed as the change in OCR/ECAR measurements following vehicle/metformin treatment, when compared to each corresponding baseline measurement. Metabolic rates were normalised to protein content using the Pierce BCA assay as per the manufacturers’ instructions.

### 2.18 Multiplex enzyme-linked immunosorbent assay profiling of TCM and NCM

To assess angiogenic, vascular injury, pro-inflammatory, cytokine and chemokine secretions a 54-plex ELISA kit separated across 7 plates was used (Meso Scale Discovery, Rockville, MD, USA). The multiplex kit was used to quantify the secretions of CRP, Eotaxin, Eotaxin-3, FGF(basic), Flt-1, GM-CSF, ICAM-1, IFN-γ, IL-10, IL-12/IL-23p40, IL-12p70, IL-13, IL-15, IL-16, IL-17A, IL-17A/F, IL-17B, IL-17C, IL-17D, IL-1RA, IL-1α, IL-1β, IL-2, IL-21, IL-22, IL-23, IL-27, IL-3, IL-31, IL-4, IL-5, IL-6, IL-7, IL-8, IL-8 (HA), IL-9, IP-10, MCP-1, MCP-4, MDC, MIP-1α, MIP-1β, MIP-3α, PlGF, SAA, TARC, Tie-2, TNF-α, TNF-β, TSLP, VCAM-1, VEGF-A, VEGF-C and VEGF-D. All assays were run as per manufacturer’s recommendation, an overnight supernatant incubation protocol was used for all assays except Angiogenesis Panel 1 and Vascular Injury Panel 2, which were run according to the same day protocol. TCM and NCM were run undiluted on all assays except Vascular Injury Panel 2, where a one-in-four dilution was used. Secretion data for all factors was normalised to cell lysate protein content by using a BCA assay.

### 2.19 Statistical analysis

All statistical analysis and graphing were performed using Graphpad Prism v9 software. Data is presented as mean ± standard error of the mean (SEM) throughout. Statistical comparisons were carried out using analysis of variance (ANOVA) testing, *post-hoc* Tukey’s multiple comparisons testing or *t*-testing, depending on the experimental set up, as described in figure legends. For transcriptomic data analysis, BlueBee, DESeq2 R extension and IPA software were utilised for statistical analysis. DESeq2 utilised Wald testing, while IPA utilised Fisher’s Exact Test, as stated in figure/table legends. Analysis on patient samples used Mann-Whitney U or Wilcoxon signed rank test, as appropriate. Results were considered significant where probability (*p)* ≤ 0.05.

## 3 Results

### 3.1 Metformin treatment significantly alters energy metabolism and mitochondrial function in HCT116 and SW837 CRC cells

To investigate the metabolic modulating effects of metformin treatment on CRC cells *in vitro*, the metabolic phenotypes of HCT116 colon cancer and SW837 rectal cancer cells treated with metformin were assessed by Seahorse profiling. This permits the measurement of the two major metabolic pathways, oxidative phosphorylation, represented by oxygen consumption rate (OCR), and glycolysis, represented by extracellular acidification rate (ECAR) in live cells in real-time.

**Figure 1:**
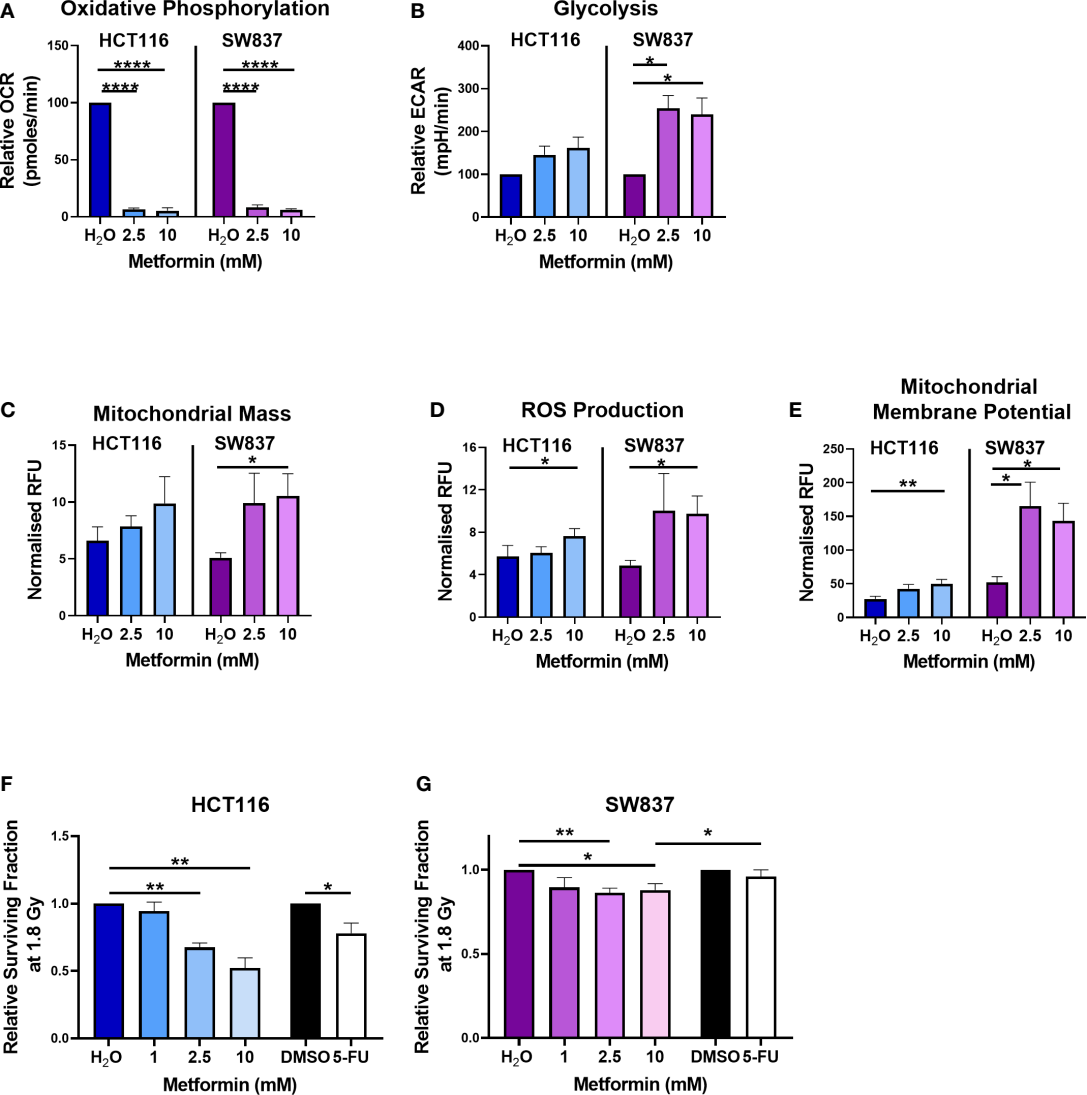
Metformin alters energy metabolism, mitochondrial function and radiosensitivity in CRC cells. HCT116 and SW837 cells were treated with metformin (2.5 mM or 10 mM) or vehicle control (H_2_O) for 24 h, and energy metabolism and mitochondrial function was assessed using Seahorse technology and fluorescent probes, respectively. **(A)** OCR **(B)** ECAR **(C)** Mitochondrial mass **(D)** ROS production **(E)** Mitochondrial membrane potential. HCT116 cells and SW837 cells were treated with metformin (1 mM, 2.5 mM, 10 mM) or H_2_O vehicle control for 24 h, or 5-FU (15 µM) or DMSO control (0.001%) for 6 h, prior to irradiation with 1.8 Gy X-ray radiation and radiosensitivity was assessed by clonogenic assay. **(F)** Surviving fraction of HCT116 cells irradiated with 1.8 Gy following pre-treatment with metformin, 5-FU or vehicle controls. **(G)** Surviving fraction of SW837 cells irradiated with 1.8 Gy following pre-treatment with metformin, 5-FU or vehicle controls. Data is presented as mean ± SEM for at least 3 independent experiments. Statistical analysis was performed using *t*-testing or ANOVA as appropriate. *p < 0.05, **p < 0.01, ****p < 0.0001.

Metformin treatment (2.5 mM and 10 mM) significantly reduced OCR in HCT116 cells, when compared to vehicle control (**Figure 1A**). In SW837 rectal cancer cells, metformin (2.5 mM and 10 mM) significantly inhibited OCR and upregulated ECAR, relative to vehicle control (**Figures 1A, B**). To further investigate the impact of metformin treatment on mitochondrial metabolism in HCT116 and SW837 cells, mitochondrial dysfunction was assessed using three surrogate markers of mitochondrial function; mitochondrial mass, reactive oxygen species (ROS) and mitochondrial membrane potential. No significant alterations to mitochondrial mass were demonstrated following metformin treatment in HCT116 cells, however, in SW837 cells, mitochondrial mass was significantly increased following 24 h treatment with metformin (10 mM), when compared to control (**Figure 1C**). ROS production was significantly increased in HCT116 and SW837 cells following treatment with 10 mM metformin for 24 h, when compared to control (**Figure 1D**). Furthermore, mitochondrial membrane potential was significantly increased in both cell lines following 24 h treatment with 10 mM metformin, when compared to control (**Figure 1E**). These data demonstrate that metformin alters metabolism, specifically inhibiting oxidative phosphorylation, and induces significant mitochondrial dysfunction in both HCT116 and SW837 cells, supporting its metabolic modulatory effects in CRC *in vitro*.

### 3.2 Metformin treatment significantly radiosensitises HCT116 and SW837 CRC cells

Having demonstrated metformin-mediated inhibition of oxidative phosphorylation in both HCT116 and SW837 cells, the potential of metformin as a radiosensitiser *in vitro* was investigated. We and others have previously identified SW837 rectal cancer and HCT116 colon cancer cells as an *in vitro* model of inherently radioresistant, and radiosensitive CRC, respectively (23, 33). The potential radiosensitising effects of metformin (1 mM, 2.5 mM, 10 mM) following a clinically-relevant dose of 1.8 Gy X-ray radiation was assessed in HCT116 and SW837 cells using the gold standard clonogenic assay.

Metformin treatment (2.5 mM and 10 mM) significantly sensitised radiosensitive HCT116 cells to 1.8 Gy radiation, when compared to vehicle control (**Figure 1F**) (relative mean surviving fraction (SF) ± standard error of mean (SEM); 2.5 mM metformin 0.67 ± 0.03, 10 mM 0.53 ± 0.08). Interestingly, this metformin-mediated radiosensitisation was superior to the radiosensitising effects of 5-FU (15 µM) (**Figure 1F**) (relative mean SF ± SEM; 15 µM 5-FU 0.78 ± 0.08). Metformin treatment (2.5 mM and 10 mM) also significantly sensitised radioresistant SW837 cells to 1.8 Gy X-ray radiation, when compared to vehicle control (**Figure 1G**) (relative mean SF ± SEM; 2.5 mM metformin 0.86 ± 0.3, 10 mM metformin 0.88 ± 0.04). Importantly, this metformin-mediated radiosensitisation of SW837 cells was significantly superior (p < 0.05) to the radiosensitising effects of 5-FU (relative mean SF ± SEM; 15 µM 5-FU 0.98 ± 0.045), the current standard of care.

These data demonstrate that metformin, significantly radiosensitises radiosensitive HCT116 cells and radioresistant SW837 cancer cells at a clinically-relevant dose of 1.8 Gy. Importantly, the radiosensitising effects of metformin on both cell lines at 1.8 Gy were superior to the radiosensitising effects demonstrated by 5-FU, the current standard of care chemotherapeutic.

### 3.3 Metformin alters cell cycle distribution and radiation-induced progression in CRC cells

Having demonstrated that metformin significantly radiosensitises HCT116 and SW837 cells, the potential mechanism(s) underlying this metformin-mediated radiosensitisation were investigated. Basal cell cycle distribution has been implicated in the radio response, with cells in the S phase and G0/G1 phases being more radioresistant, and cells in the G2/M phase being the most sensitive to radiation exposure (34).

**Figure 2:**
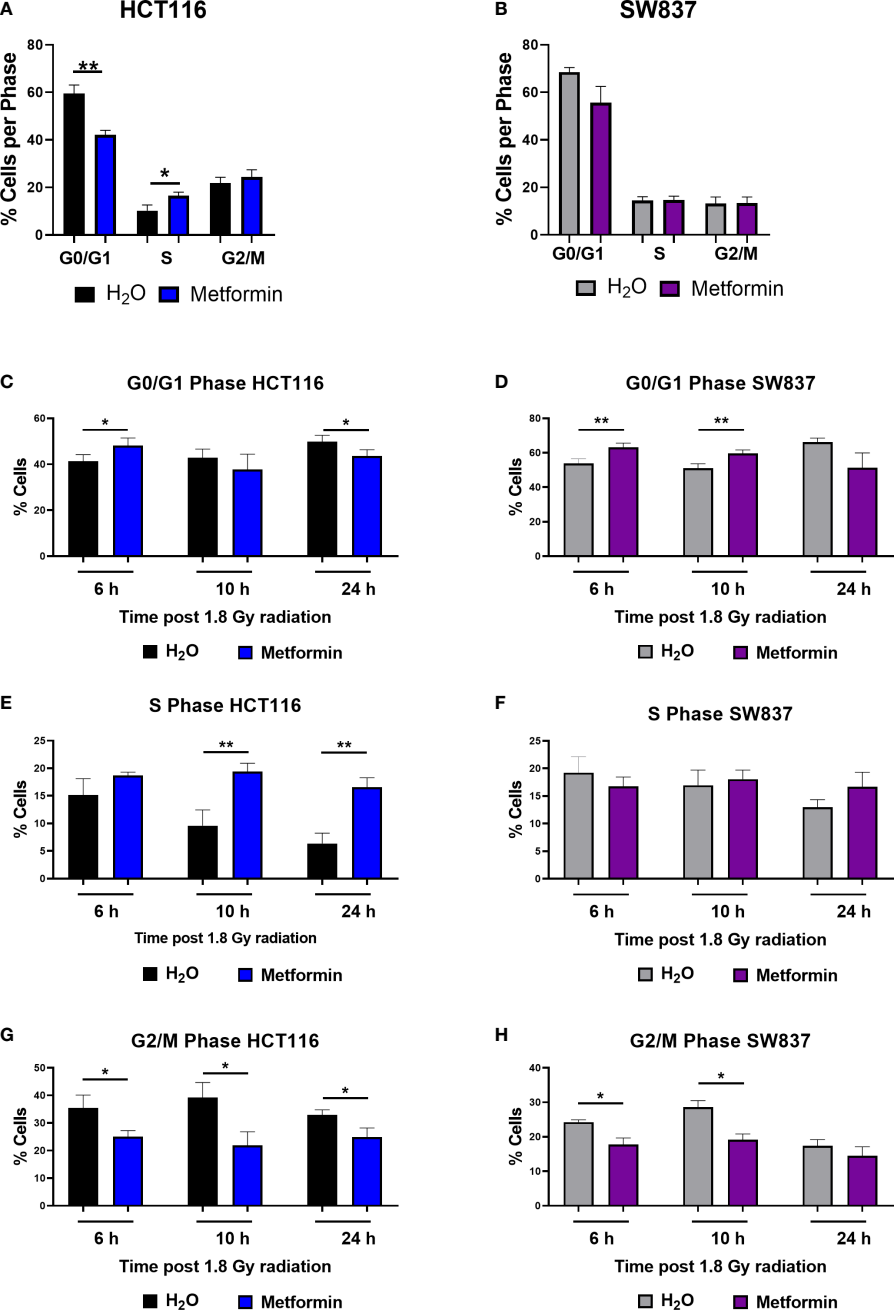
Metformin significantly alters basal cell cycle distribution and radiation-induced cell cycle progression in CRC cells. HCT116 and SW837 cells were treated with metformin (10 mM) or H_2_O vehicle control for 24 h before irradiation with 1.8 Gy X-ray radiation. Controls were mock irradiated. Cell cycle distribution was assessed basally and at 6 h, 10 h and 24 h post irradiation by PI staining and flow cytometry. **(A)** Basal cell cycle in HCT116 cells following treatment (48 h) with metformin or vehicle control. **(B)** Basal cell cycle in SW837 cells following treatment (48 h) with metformin or vehicle control. Proportion of G0/G1 phase cells following metformin and radiation treatment in **(C)** HCT116 and **(D)** SW837 cells. Proportion of S phase cells following metformin and radiation treatment in **(E)** HCT116 and **(F)** SW837 cells. Proportion of G/M phase cells following metformin and radiation treatment in **(G)** HCT116 and **(H)** SW837 cells. Data is presented as mean ± SEM for at least 4 independent experiments. Statistical analysis was performed using paired *t*-testing. *p < 0.05, **p < 0.01.

Metformin treatment significantly reduced the proportion of radiosensitive HCT116 cells in the G0/G1 phase at 48 h post treatment, when compared to vehicle control (**Figure 2A**). By 48 h post treatment, metformin treatment resulted in a significant increase to the proportion of HCT116 cells in S phase (**Figure 2A**). In contrast, in radioresistant SW837 cells, no significant alterations in cell cycle distribution were observed at 48 h post treatment in SW837 cells (**Figure 2B**). These data demonstrate differential effects of metformin treatment on basal cell cycle distribution in HCT116 and SW837 cells.

Given the demonstrated metformin-mediated alteration in basal cell cycle distribution in HCT116 cells, the effect of metformin on cell cycle progression following irradiation with X-ray radiation was investigated in HCT116 and SW837 cells. HCT116 and SW837 cells were pre-treated with metformin (10 mM) or H_2_O vehicle control for 24 h, exposed to 1.8 Gy radiation, and the cell cycle distribution assessed at 6 h, 10 h and 24 h post irradiation.

In radiosensitive HCT116 cells, the addition of metformin to 1.8 Gy X-ray radiation significantly increased the proportion of G0/G1 cells at 6 h post irradiation, but by 24 h post irradiation, the proportion of G0/G1 cells were significantly reduced in cells treated with metformin and 1.8 Gy, when compared to irradiated vehicle control (**Figure 2C**). The addition of metformin to 1.8 Gy significantly increased the proportion of S phase HCT116 cells at 10 h and 24 h post irradiation, when compared to irradiated vehicle controls (**Figure 2E**). The addition of metformin to 1.8 Gy significantly reduced the proportion of HCT116 G2/M phase cells at 6h, 10 h and 24 h post radiation, when compared to irradiated vehicle controls (**Figure 2G**).

In radioresistant SW837 cells, the addition of metformin to 1.8 Gy induced a significant G0/G1 arrest at 6 h and 10 h post irradiation, when compared to irradiated vehicle control (**Figure 2D**). At all timepoints post radiation, no significant differences were demonstrated in the proportion of S phase SW837 cells (**Figure 2F**). Metformin significantly reduced the proportion of SW837 cells in G2/M phase at both 6 h and 10 h post irradiation with 1.8 Gy, when compared to irradiated vehicle control (**Figure 2H**).

Together, these data demonstrate that metformin treatment in combination with radiation induces significant alterations to cell cycle progression, with differing effects on the radiosensitive HCT116 and radioresistant SW837 cell lines.

### 3.4 Metformin significantly induces cell death basally and following treatment with X-ray radiation in CRC cells

As metformin has been demonstrated to exert anti-cancer effects, we investigated if metformin-induced apoptosis may be a mechanism underlying the metformin-mediated radiosensitisation of HCT116 and SW837 cells.

**Figure 3:**
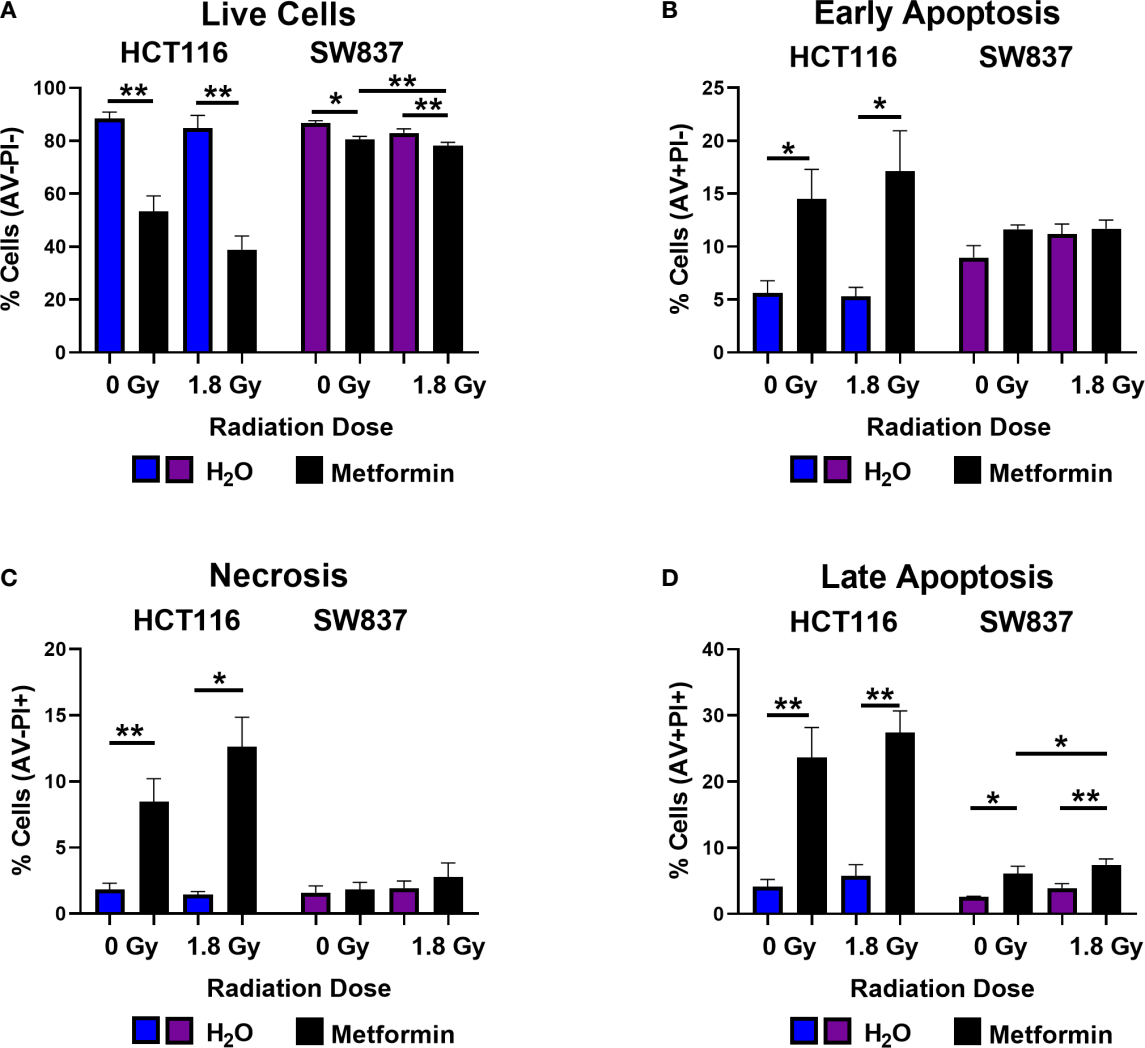
Metformin induces cell death in HCT116 and SW837 cells basally and following X-ray radiation. HCT116 and SW837 cells were treated with metformin (10 mM) or H_2_O vehicle control for 24 h, either mock-irradiated or exposed to 1.8 Gy X-ray radiation, and cell death was assessed by Annexin V/PI staining and flow cytometry at 48 h post irradiation/72 h post metformin treatment. **(A)** Live cells. **(B)** Early apoptotic cells. **(C)** Necrotic cells. **(D)** Late Apoptotic cells. Data is presented as mean ± SEM for 5 independent experiments. Statistical analysis was performed by paired t-testing. *p<0.05, **p< 0.01.

In unirradiated cells, metformin treatment (72 h) significantly reduced the number of live HCT116 and SW837 cells, when compared to vehicle control (**Figure 3A**). Metformin treatment (72 h) induced a significant increase in early and late apoptotic HCT116 cells, when compared to vehicle control (**Figures 3B, D**). In addition, metformin significantly induced significant late apoptosis in SW837 cells, when compared to vehicle control (**Figure 3D**). Furthermore, metformin significantly induced necrosis in HCT116 cells, when compared to vehicle control (**Figure 3C**). Together, these data demonstrate that metformin treatment (10 mM) induces significant cell death in HCT116 and SW837 CRC cell lines.

Having demonstrated that metformin treatment alone induces cell death in both HCT116 and SW837 cells, the impact of metformin combined with a clinically-relevant dose of 1.8 Gy radiation was assessed at 48 h post radiation exposure. Metformin treatment significantly reduced the percentage of live HCT116 and SW837 cells following irradiation with 1.8 Gy, when compared to irradiated vehicle controls (**Figure 3A**). Furthermore, combination metformin and 1.8 Gy radiation treatment significantly increased the percentage of early apoptotic HCT116 cells, when compared to irradiated vehicle control (**Figure 3B**). Necrotic cells were also significantly increased at 48 h post radiation in HCT116 cells treated with combination metformin and 1.8 Gy radiation, when compared to the irradiated vehicle control (**Figure 3C**). Combining metformin treatment with 1.8 Gy radiation also significantly increased the percentage of late apoptotic cells, when compared to irradiated vehicle controls in both HCT116 and SW837 cells (**Figure 3D**). Furthermore, metformin combined with 1.8 Gy radiation significantly increased the proportion of SW837 cells in late apoptosis, when compared to unirradiated metformin treated cells (**Figure 3D**). Together, these data demonstrate that metformin induces cell death in HCT116 and SW837 alone and in combination with radiation treatment, and that may be a contributing mechanism underlying the metformin-mediated radiosensitisation of HCT116 and SW837 cells.

### 3.5 Metformin significantly increases total glutathione production in CRC cells *in vitro*

As the majority of DNA damage induced by radiation is via indirect action, in particular oxidative damage, and given the demonstrated metformin-induced ROS production in HCT116 and SW837 cells (**Figure 1D**), the impact of metformin treatment on anti-oxidant capacity was assessed. Total glutathione, measuring both reduced glutathione (GSH) and oxidised glutathione (GSSG) was assessed in metformin treated HCT116 and SW837 cells by luminescent assay.

**Figure 4:**
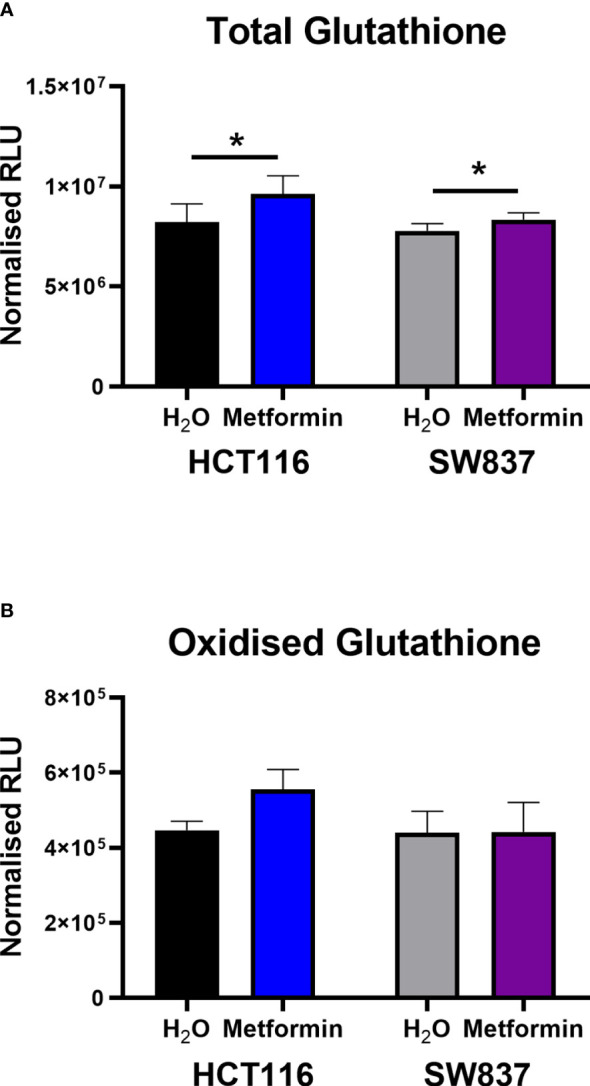
Metformin significantly increases GSH levels in HCT116 and SW837 cells. GSH and GSSG levels were assessed by GSSG/GSH-Glo™ luminescent assay in HCT116 and SW837 cells at 24 h post treatment with either metformin (10mM) or H_2_O vehicle control. **(A)** Total GSH levels in HCT116 and SW837 cells. **(B)** GSSG levels in HCT116 and SW837 cells. Data is presented as mean ± SEM for *n*=5 (HCT116) or *n*=6 (SW837) independent experiments. Statistical analysis was performed by paired t-testing *p < 0.05.

Metformin treatment significantly increased total glutathione production in both HCT116 and SW837 cells, when compared to vehicle control (**Figure 4A**). Interestingly, when measuring the levels of GSSG alone, no significant alterations were demonstrated in either HCT116 or SW837 cells following metformin treatment (**Figure 4B**). Together, this data demonstrates that metformin significantly affects total glutathione production, suggesting metformin-mediated alteration of the redox balance in CRC cells.

### 3.6 Metformin treatment significantly alters the transcriptome of SW837 cells

To further investigate potential mechanisms underlying metformin-mediated radiosensitisation, transcriptomic profiling was performed on radioresistant SW837 cells following 24 h treatment with metformin (10 mM).

**Figure 5:**
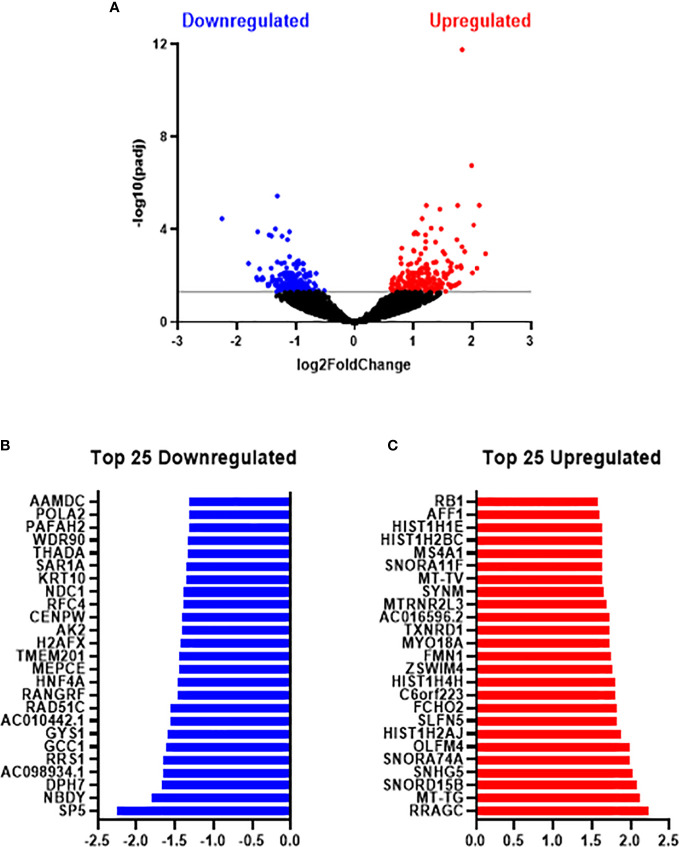
Metformin significantly alters the transcriptome of radioresistant SW837 cells. SW837 cells were treated with Metformin (10 mM) or H_2_O vehicle control for 24 h and transcriptomics was performed using the Lexogen QuantSeq 3’ mRNA-Seq sequencing platform. **(A)** Volcano plot demonstrating 407 genes significantly altered in SW837 cells treated with metformin, when compared to H_2_O vehicle control. The y-axis corresponds to the -log10(*p*-adj), and the x-axis represents the Log2 (Fold Change). Dots in blue and red represent the significantly downregulated/upregulated genes in metformin treated SW837 cells. Dots in black represent the genes that did not reach statistical significance (*p*-adj > 0.05). **(B)** The top 25 downregulated genes (by fold change) in SW837 cells treated with metformin, when compared to H_2_O vehicle control. **(C)** The top 25 upregulated genes (by fold change) in metformin treated SW837 cells, when compared to H_2_O vehicle control. Data is presented for 4 independent experiments. Statistical analysis was performed using the Wald test, with corrections for multiple comparisons performed by the Benjamini-Hochberg correction (FDR).

In total, 24,391 genes were expressed across metformin and vehicle control treated SW837 cells. Differential expression analysis identified 417 genes significantly altered between SW837 cells treated with metformin and vehicle control, based on adjusted *p*-value (*p*-adj) < 0.05 (**Figure 5A**), with 200 genes significantly downregulated, and 217 genes significantly upregulated in metformin treated SW837 cells, when compared to vehicle control (**Figure 5A**). Of the significantly altered genes, the top 25 downregulated and upregulated genes in metformin treated SW837 cells are demonstrated in **Figures 5B, C**.

Having demonstrated significant alterations in the transcriptome of metformin treated SW837 cells, the specific biological pathways in which these altered genes are involved, were assessed using IPA. These data demonstrate significant alterations to molecular and cellular functions in SW837 cells treated with metformin, including ‘cell cycle’ and ‘Cell Death and Survival’ (**Table 1**).

**Table 1:** Significantly altered biological functions in metformin treated SW837 cells.

Category	*p*-value range
Cellular Assembly and Organization	9.26E-07-6.84E-04
Cellular Function and Maintenance	9.26E-07-7.22E-04
Cell Cycle	6.64E-06-4.98E-04
DNA Replication, Recombination, and Repair	6.64E-06-8.3E-05
Protein Synthesis	7.08E-05-7.29E-04
Cell Death and Survival	2.81E-04-5.56E-04
Cellular Movement	2.83E-04-3.54E-04
Cell Signalling	4.68E-04-4.68E-04
Post-Translational Modification	4.68E-04-4.68E-04
Molecular Transport	5.76E-04-5.76E-04
RNA Trafficking	5.76E-04-5.76E-04
Gene Expression	6.37E-04-6.37E-04
Cell-To-Cell Signalling and Interaction	7.22E-04-7.22E-04
Cellular Compromise	7.22E-04-7.22E-04

Biostatistical analysis was performed on significantly altered genes in metformin treated SW837 cells, when compared to H_2_O vehicle control by IPA analysis to identify predicted altered biological functions. Statistical analysis was performed by right-tailed Fisher’s exact test using IPA analysis.

To further interrogate the specific pathways involved in the altered biological functions in metformin treated SW837 cells, Canonical Pathway Analysis was also performed on differentially expressed genes using IPA software. The top 10 most significantly altered canonical pathways are demonstrated in **Table 2** (with all significantly altered pathways displayed in **Supplementary Table S1**). Interestingly, IPA software predicted an inhibition of oxidative phosphorylation in metformin treated SW837 cells, supporting the demonstrated metformin-induced inhibition of OCR using Seahorse live-cell metabolic profiling (**Figure 1**). Of the ten significantly altered oxidative phosphorylation genes in metformin treated SW837 cells, six were Complex I genes, supporting metformin as a complex I inhibitor in rectal cancer. In addition, ‘mitochondrial dysfunction’ was also demonstrated as a significantly altered canonical pathway following metformin treatment, further supporting our functional analyses. Together these data support metformin as a metabolic modulator in rectal cancer. In addition, many of the significantly altered canonical pathways in metformin treated SW837 cells are associated with the cellular radioresponse, including cell cycle, cell death and hypoxic signalling.

**Table 2:** Top 10 most significantly altered canonical pathways in metformin treated SW837 cells.

Ingenuity Canonical Pathways	-log(*p*-value)
Sirtuin Signalling Pathway	5.08
Oxidative Phosphorylation	4.49
Mitochondrial Dysfunction	4.14
Huntington’s Disease Signalling	4.08
Remodelling of Epithelial Adherens Junctions	3.62
Germ Cell-Sertoli Cell Junction Signalling	3.49
Hypoxia Signalling in the Cardiovascular System	3.31
FAT10 Signalling Pathway	3.29
Oestrogen Receptor Signalling	3.19
Unfolded protein response	2.84

### 3.7 Metformin significantly inhibits oxidative phosphorylation and glycolysis in rectal adenocarcinoma biopsies

Having demonstrated metabolic modulatory effects of metformin in an *in vitro* model of rectal cancer, the effect of metformin treatment on the metabolic rate of treatment naïve *ex vivo* rectal adenocarcinoma biopsies was assessed in real-time using the Seahorse XFe analyser. Rectal tumour biopsies (*n*=10) were collected from consenting patients undergoing diagnostic endoscopy, a baseline measurement of metabolic rate was recorded, biopsies were treated for 24 h with metformin (10 mM) or H_2_O vehicle control and metabolism was assessed at 24 h post treatment. The patient cohort characteristics are outlined in **Table 3**.

**Table 3:** Patient characteristics of patient cohort used in *ex vivo* real time metabolic profiling.

		Cancers (*n*=10)	Non-Cancers (*n*=12)
**Gender**	**Male (*n*)**	5	6
	**Female (*n*)**	5	6
**Age at diagnosis**	**Median (range)**	69 (47–78)	41.5 (26–81)
Clinical T stage*	**2 (*n*)**	3	
	**3 (*n*)**	5	
	**4 (*n*)**	1	
Clinical N stage*	**0 (*n*)**	6	
	**2 (*n*)**	2	
	**3 (*n*)**	1	
**Differentiation Stage**	**Moderate-poor (*n*)**	1	
	**Moderate (*n*)**	5	
	**Well (*n*)**	1	
	**Unknown (*n*)**	3	

*clinical T stage, clinical N stage only available for *n* = 9 patients. T stage, tumour stage; N stage, nodal stage.

**Figure 6:**
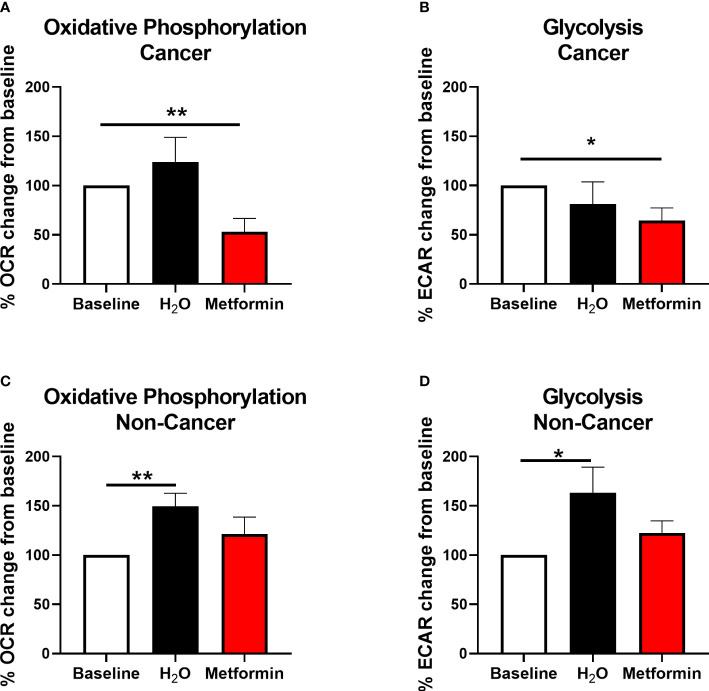
Metformin alters metabolism in rectal cancer tissue biopsies. OCR and ECAR were measured in treatment naïve rectal cancer biopsies or normal, non-cancer rectal tissue prior to and 24 h post treatment with metformin (10 mM) or H_2_O vehicle control using the Seahorse XFe24 analyser. **(A)** OCR percentage change from baseline following treatment with metformin or H_2_O vehicle control in rectal cancer biopsies. **(B)** ECAR percentage change from baseline following treatment with metformin or H_2_O vehicle control in rectal cancer biopsies. **(C)** OCR percentage change from baseline following treatment with metformin or H_2_O vehicle control in normal, non-cancer rectal tissue. **(D)** ECAR percentage change from baseline following treatment with metformin or H_2_O vehicle control in normal, non-cancer rectal tissue. Data is normalised to protein content and presented as mean ± SEM. *n* =10 (cancer) or *n* = 12 (non-cancer). Statistical analysis was performed using Wilcoxon matched pairs signed rank test. *p < 0.05, **p< 0.01.

Metformin significantly inhibited OCR by 47% (**Figure 6A**) and ECAR by 36% (**Figure 6B**) in rectal adenocarcinoma biopsies, when compared to baseline, demonstrating metformin-induced modulation of energy metabolism in rectal tumours from patients.

Having demonstrated that metformin treatment significantly inhibits oxidative phosphorylation in both *in vitro* and *ex vivo* models of rectal cancer, the effect of metformin on histologically-confirmed normal rectal tissue biopsies (*n*=12, median age of 41.5 y) taken from non-cancer patients was assessed in real-time using the Seahorse XFe analyser. Whilst significant alterations in OCR and ECAR were demonstrated in normal rectal tissue biopsies treated with H_2_O vehicle control versus baseline, interestingly, no significant alterations in OCR or ECAR was demonstrated following metformin treatment, when compared to baseline (**Figures 6C, D**).

This suggests that metformin treatment alters energy metabolism in rectal adenocarcinoma tissue, but not in non-cancer rectal tissue biopsies, supporting its potential utility as a radiosensitiser in rectal cancer.

### 3.8 Metformin significantly alters the inflammatory secretome of rectal cancer and normal rectal tissue

A key process influencing metabolism in the tumour microenvironment is inflammation. Therefore, to further assess the effect of metformin on both rectal cancer and normal, non-cancer rectal tissue, the protein secretome of treatment naïve rectal tumour biopsies and non-cancer biopsies was assessed following metformin treatment. Tumour conditioned media (TCM) and normal, non-cancer conditioned media (NCM) samples from biopsies treated with either metformin or H_2_O vehicle control were profiled for inflammatory, angiogenic, chemokine and cytokine secretions using the MSD 54 multiplex ELISA (**Supplementary Table S2**). Patient cohort characteristics are outlined in **Table 4**.

**Table 4:** Patient characteristics of the rectal cancer patient cohort utilised in multiplex ELISA secretome profiling.

		Cancers (*n*=12)	Non-Cancers (*n*=12)
**Gender**	**Male (*n*)**	6	6
	**Female (*n*)**	6	6
**Age at diagnosis**	**Median (range)**	69 (47–78)	41.5 (26–81)
Clinical T stage*	**1/2 (*n*)**	1	
	**2 (*n*)**	3	
	**3 (*n*)**	6	
	**4 (*n*)**	1	
Clinical N stage*	**0 (*n*)**	7	
	**2 (*n*)**	3	
	**3 (*n*)**	1	
**Differentiation stage**	**Moderate-poor (*n*)**	2	
	**Moderate (*n*)**	6	
	**Well (*n*)**	1	
	**Unknown(*n*)**	3	

*clinical T stage, clinical N stage only available for *n* = 11 patients. T stage, tumour stage; N stage, nodal stage.

**Figure 7:**
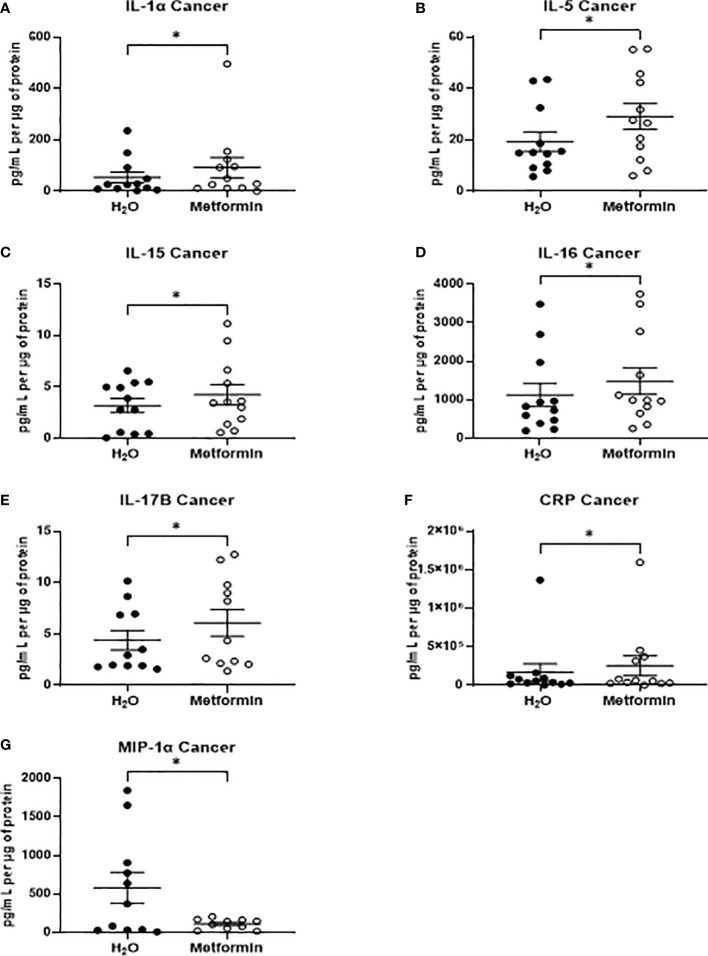
Metformin alters the inflammatory secretome of rectal cancer tissue biopsies. Treatment naïve rectal cancer tissue biopsies were treated with metformin (10 mM) or H_2_O vehicle control for 24h, TCM was collected and profiled using multiplex ELISA. **(A)** IL-1α **(B)** IL-5 from **(C)** IL-15 **(D)** IL-16, **(E)** IL-17B **(F)** CRP and **(G)** MIP-1α were significantly altered in TCM following metformin treatment. Data is normalised to protein content and presented from *n*=12 patient samples. Statistical analysis was performed by Wilcoxon signed rank *t*-test. *p < 0.05.

In TCM samples, 7 proteins were demonstrated to be significantly altered in metformin treated samples, when compared to vehicle control (**Figures 7A–G**). Of these, five proteins were cytokines (Interleukin (IL)-1α, IL-5, IL-15, IL-16 IL-17B) the secretion of which were significantly increased following metformin treatment, when compared to vehicle control (**Figures 7A–E**). Secreted levels of C-reactive protein (CRP) were significantly increased in the secretome of metformin treated TCM, when compared to vehicle control (**Figure 7F**). Macrophage inflammatory protein 1α (MIP-1α), a chemokine, was significantly decreased in metformin treated TCM, when compared to vehicle control (**Figure 7G**).

**Figure 8:**
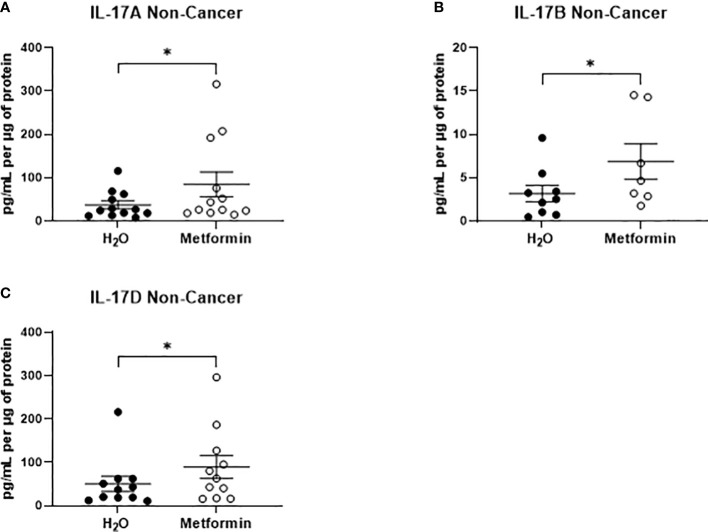
Metformin significantly alters the secretion of IL-17 related cytokines from non-cancer rectal tissue. Normal, non-cancer rectal tissue biopsies were treated with metformin (10 mM) or H_2_O vehicle control for 24h, NCM was collected and profiled using multiplex ELISA. **(A)** IL-17A, **(B)** IL-17B and **(C)** IL-17D were significantly altered in NCM treated with metformin. Data is normalised to protein content and presented from *n*=12 patient samples. Statistical analysis was performed by Wilcoxon signed rank *t*-test. *p < 0.05.

In NCM samples, the secretion of only three inflammatory factors were significantly increased from non-cancer rectal tissue treated with metformin, when compared to vehicle control (**Figures 8A–C**). All three significantly altered factors were cytokines in the IL-17 family (IL-17A, IL-17B, IL-17D) (**Figures 8A–C**). These data demonstrate that metformin treatment differentially alters the inflammatory secretome of rectal cancer tissue and non-cancer rectal tissue.

## 4 Discussion

Resistance to the standard of care, neoCRT, is a major clinical challenge in the management of rectal cancer. There is an urgent unmet need to develop novel therapeutic approaches to enhance the tumour response to neoCRT to offer improved treatment and survival rates for rectal cancer patients.

Increasing evidence supports a role for altered metabolism in the tumour response to treatment (18, 21, 22, 35–41). We have previously demonstrated that enhanced oxidative phosphorylation and reduced dependence on glycolysis is associated with a radioresistant phenotype in oesophageal adenocarcinoma (21, 22) and rectal adenocarcinoma (23), respectively, suggesting that targeting energy metabolism, specifically oxidative phosphorylation, may be an effective strategy to enhance therapeutic response to neoCRT (42).

Metformin, which is approved clinically for the management of type II diabetes, has an elusive precise mechanism of action, however, it has been demonstrated to inhibit complex I of the ETC (24, 25). Recently, data has emerged supporting the potential utility of metformin as a radiosensitiser in multiple cancers (43–46). In observational studies, rectal cancer patients with diabetes treated with metformin during their cancer treatment, either neoCRT or peri-operative chemotherapy, have demonstrated higher rates of pCR, when compared to those patients not receiving metformin (27). Furthermore, metformin treatment is demonstrated to enhance radiosensitivity *in vitro* and in xenograft models of colon cancer (30, 31) and pancreatic cancer (47). However, the precise role of metformin as an anti-metabolic radiosensitiser in rectal cancer is largely unknown.

In this study, SW837 rectal cancer cells, and HCT116 colon cancer cells were utilised as an *in vitro* model of inherently radioresistant, and radiosensitive CRC, respectively. We and others have previously characterised these *in vitro* models of CRC, demonstrating SW837 rectal cells as a robust model of radioresistant rectal cancer (23, 48, 49). We have previously demonstrated that SW837 cells have a reduced reliance on glycolysis, supporting a role for enhanced mitochondrial metabolism in the radioresistance of these cells (23). In this study, metformin was demonstrated to significantly inhibit oxidative phosphorylation and enhance glycolytic rates in both HCT116 and SW837 cells, supporting it as an ETC inhibitor in CRC. Metformin was also demonstrated to alter mitochondrial function in CRC, specifically increasing mitochondrial mass in SW837 cells and altering ROS production and mitochondrial membrane potential in SW837 and HCT116 cells, supporting previous studies in models of prostate, endometrial, pancreatic, lung, breast and CRC (50–54). Furthermore, these functional analyses were supported by significant alterations to oxidative phosphorylation and mitochondrial function demonstrated through transcriptomic profiling of metformin-treated SW837 rectal cancer cells. In particular, 6 of the 10 oxidative phosphorylation genes demonstrated to be altered in SW837 cells were genes of Complex I, supporting the role of metformin as a Complex I inhibitor in rectal cancer. Together, this data supports metformin as a ETC complex I inhibitor in radioresistant rectal cancer.

In addition to modulation of energy metabolism and mitochondrial function in CRC, we demonstrate for the first time, that metformin significantly radiosensitises both radiosensitive HCT116 colon cancer and radioresistant SW837 rectal cancer cells to a clinically-relevant dose of 1.8 Gy X-ray radiation. Importantly, this metformin-mediated radiosensitisation was superior to that of 5-FU, which is the current standard of care. This supports previous studies demonstrating metformin-mediated radiosensitisation of HCT116 colon cancer cells (30, 55, 56) and importantly highlights the potential utility of metformin as a novel radiosensitiser in rectal cancer.

The precise mechanisms underpinning the observed radiosensitising effects of metformin treatment in cancer are poorly understood. This study demonstrates that metformin inhibits oxidative phosphorylation in CRC cells, supporting metformin-mediated inhibition of complex I as a potential mechanism underlying its radiosensitising effects. Interestingly, novel metformin analogues (e.g. Mito-Met_10_), with enhanced mitochondrial targeting capacity, have been demonstrated to enhance metformin-mediated inhibition of mitochondrial respiration and radiosensitisation in prostate cancer (57), suggesting that metformin-mediated modulation of mitochondrial metabolism is a key mechanism underlying its radiosensitising effects. Other inhibitors of oxidative phosphorylation have been demonstrated to induce radiosensitisation in various cancer types. Our department recently demonstrated that Pyrazinib, a small molecule drug, inhibits oxidative phosphorylation in both *in vitro* and *ex vivo* models of oesophageal adenocarcinoma (21, 58). Furthermore, treatment with this oxidative phosphorylation inhibitor drug was demonstrated to significantly radiosensitise radioresistant oesophageal adenocarcinoma cells to clinically-relevant doses of X-ray radiation (21). In a recent study conducted by Lan et al., significant radiosensitisation was demonstrated to be induced by a series of oxidative phosphorylation inhibitor drugs in 3D models of colon, breast and cervical cancer cell lines (59). Together, these data support the data presented here suggesting that inhibition of oxidative phosphorylation is a potential mechanism underlying metformin-mediated radiosensitisation in CRC.

We also demonstrate metformin-mediated alterations to cell cycle distribution and progression following radiation in HCT116 and SW837 cells, with metformin demonstrated to reduce the proportion of G2/M phase cells and prevent radiation-induced G2/M blockade in these cells. In cancer cells, G2/M arrest is a common response following exposure to ionising radiation, to permit the repair of double-strand breaks. Importantly, elimination of this radiation-induced G2/M blockade is a promising radiosensitising approach as cancer cells frequently have a deficient G1 checkpoint (60, 61). This reduction in G2/M phase cells following metformin treatment has been previously demonstrated in HCT116 cells (62), however, this is the first time this has been demonstrated in radioresistant SW837 rectal cancer cells. Furthermore, metformin treatment alone was demonstrated to significantly induce cell death in both HCT116 and SW837 cells, although this effect was more pronounced in HCT116 cells, supporting previous studies (33, 63). Metformin treatment has previously been demonstrated to induce apoptosis in multiple cancer types (33, 64–67), supporting these findings. Importantly, combination of metformin and radiation significantly increased cell death, when compared to irradiated vehicle controls, highlighting the potential role for altered cell death in the metformin-mediated radiosensitisation of these cell lines. Together, this functional data indicates that inhibition of oxidative phosphorylation, cell cycle alterations, and mitigation of radiation-induced G2/M arrest may contribute to metformin-mediated radiosensitisation in CRC. Importantly, this was supported by transcriptomic profiling, which demonstrated significant metformin-induced alterations to a number of mechanisms implicated in the cellular radioresponse, including cell cycle, cell death and survival. Metformin was also demonstrated to alter expression of genes involved in metabolism, specifically inhibition of oxidative phosphorylation genes, supporting functional analyses and highlighting metformin as a metabolic modulator in rectal cancer. Importantly, this transcriptomic analysis demonstrated that 6 genes significantly altered in metformin-treated SW837 cells are complex I genes, a known target of metformin treatment, further supporting oxidative phosphorylation as a metabolic target of metformin treatment in rectal cancer. To our knowledge, this is the first study to perform transcriptomic profiling on metformin treated SW837 rectal cancer cells, and supports a previous study in colon cancer cells, demonstrating metformin-induced alteration of genes involved in reactive oxygen species production, cell cycle, programmed cell death and energy metabolism pathways (68).

Interestingly, many of the pathways identified in this study to be altered by metformin in both functional and transcriptomic analysis have been previously implicated in the cellular radioresponse. Cell cycle distribution significantly affects cellular radiosensitivity, with cells in each phase demonstrating different inherent radiosensitivities (34). Furthermore, progression through the cell cycle following radiation exposure is a demonstrated indicatory of cellular radiosensitivity, with G2/M arrest associated with inherent radiosensitivity (69, 70). Importantly, many of the pathways demonstrated to be altered by metformin treatment in this study, including cell cycle and cell death are dependent on cellular metabolism, indicating a potential role for altered energy metabolism as a master regulator of radioresistance in rectal cancer.

Whilst this study primarily focused on metformin-mediated effects in tumour cells, increasing evidence implicates a role for metformin in the modulation of immune cells, with metformin demonstrated to modulate macrophage polarisation (71) and the infiltration and function of T-cells (72, 73). The importance of the tumour microenvironment in the progression and treatment response of CRC is well documented, with high infiltration of T-cells associated with improved prognosis (74), suggesting that in addition to tumour cell-specific effects, metformin-mediated modulation of host immunity may also underlie its radiosensitising effects.

Importantly, the metformin-mediated metabolic alterations demonstrated in rectal cancer *in vitro*, were also supported *in vivo*, with metformin demonstrated to significantly inhibit oxidative phosphorylation and glycolysis in treatment naïve rectal cancer tissue biopsies. *Ex vivo* rectal tumour samples more accurately reflect the dynamic tumour microenvironment, the complex architecture of the tumour, and the inherent genetic diversity between patients and therefore, this data supports metformin as a metabolic modulator in rectal tumours. A key consideration for the clinical utility of radiosensitising agents is the concept of increasing the radiosensitivity of tumours whilst minimising the effects on surrounding normal tissues to achieve a high therapeutic index. In this study, metformin did not alter oxidative phosphorylation or glycolysis in normal non-cancer rectal tissue biopsies, importantly suggesting that the anti-metabolic effects of metformin may be tumour tissue-specific, which supports its potential utility as a radiosensitiser in rectal cancer.

Metabolism and inflammation are intrinsically linked and interdependent within the tumour microenvironment (75). Metformin was demonstrated to significantly alter the secretion of seven proteins from rectal cancer tissue. IL-15, which is involved in the activation and maturation of anti-tumour T cells, including natural killer (NK) cells and CD8+ T cells (76), was demonstrated to be significantly increased following metformin treatment. Interestingly, in breast cancer, the combination of IL-15 and radiation has been recently demonstrated to enhance response to radiation, improve survival and enhance CD8+ T cell infiltration (77), suggesting that this metformin-mediated increase in IL-15 secretion may in part mediate the radiosensitising effect of metformin. Furthermore, a recent study conducted by Tojo et al. demonstrated that metformin can induce the abscopal effect following radiation treatment in a murine model of lung cancer, supporting the potential importance of immune effects in combined metformin and radiation treatment (78). In contrast, in normal non-cancer rectal tissue, metformin significantly altered the secretion of only three IL-17 related cytokines, suggesting that metformin differentially alters the inflammatory secretome of normal rectal tissue, when compared to rectal cancer tissue, which may support its potential utility as a radiosensitiser.

Energy metabolism and metabolic flux are key requirement for all pathways implicated in the cellular radioresponse and suggests that metabolic reprogramming may play a central role in modulating the tumour response to anti-cancer therapy. Consequently, targeting metabolism may provide an effective strategy to boost response to anti-cancer therapy. The well-defined safety profile and demonstrated radiosensitising effects of metformin highlights its potential repurposing as a strategy to boost response to radiation therapy in cancer. Further pre-clinical and clinical studies are required to identify the optimum scheduling and determine the precise molecular mechanisms underlying metformin mediated radiosensitisation.

This study demonstrates for the first time, that metformin alters energy metabolism both *in vitro* and in *ex vivo* rectal tumour models. We demonstrate that metformin radiosensitises radioresistant rectal cancer cells to a clinically relevant dose of X-ray radiation potentially via alteration of energy metabolism, mitochondrial function, cell cycle distribution and cell death, supporting its potential as a radiosensitising agent. Importantly, this study also demonstrates limited impacts of metformin treatment on the metabolic profile of non-cancer rectal tissue, supporting its potential utility as a radiosensitising agent in the management of rectal cancer. This study highlights the need for further investigation into metformin as a novel radiosensitising agent in the treatment of rectal cancer.

## Data availability statement

Datasets are available on request: The raw data supporting the conclusions of this article will be made available by the authors, without undue reservation.

## Ethics statement

The studies involving human participants were reviewed and approved by Joint St. James’s Hospital/AMNCH ethical review board (Reference 10/11/2011) and Beacon Hospital Research Ethics Committee (Reference BEA0139). The patients/participants provided their written informed consent to participate in this study.

## Author contributions

Study Design: CB, JO’S, and NL-L; Data curation: CB, RO’B, TN, and ND; Collection of clinical samples: TN, ND, AH, DO’R, RH, PN, RK, BM, PM, CD, MK, and JL; Performed Experiments: CB; Data analysis: CB, FO’C, NL-L, and JO’S; Funding acquisition: NL-L. Manuscript Writing and Revision: CB, JR, JO’S, and NL-L. All authors contributed to the article and approved the submitted version.

## References

[1] Sung H Ferlay J Siegel RL Laversanne M Soerjomataram I Jemal A. Global cancer statistics 2020: GLOBOCAN estimates of incidence and mortality worldwide for 36 cancers in 185 countries. CA Cancer J Clin (2021) 71(3):209–49. doi: 10.3322/caac 33538338

[2] National Cancer Registry Ireland (NCRI). Cancer Incidence Projections for Ireland 2020-2045. Cork, Ireland: National Cancer Registry (2019).

[3] Araghi M Soerjomataram I Bardot A Ferlay J Cabasag CJ Morrison DS. Changes in colorectal cancer incidence in seven high-income countries: a population-based study. Lancet Gastroenterol Hepatol (2019) 4(7):511–8. doi: 10.1016/S2468-1253(19)30147-5 PMC7617144 31105047

[4] Vuik FE Nieuwenburg SA Bardou M Lansdorp-Vogelaar I Dinis-Ribeiro M Bento MJ. Increasing incidence of colorectal cancer in young adults in Europe over the last 25 years. Gut (2019) 68(10):1820–6. doi: 10.1136/gutjnl-2018-317592 PMC6839794 31097539

[5] Ullah MF Fleming CA Mealy K. Changing trends in age and stage of colorectal cancer presentation in Ireland - From the nineties to noughties and beyond. Surgeon (2018) 16(6):350–4. doi: 10.1016/j.surge.2018.03.006 29680182

[6] Saad El Din K Loree JM Sayre EC Gill S Brown CJ Dau H. Trends in the epidemiology of young-onset colorectal cancer: a worldwide systematic review. BMC Cancer (2020) 20(1):288. doi: 10.1186/s12885-020-06766-9 32252672 PMC7137305

[7] Campos FG. Colorectal cancer in young adults: A difficult challenge. World J Gastroenterol (2017) 23(28):5041–4. doi: 10.3748/wjg.v23.i28.5041 PMC5537173 28811701

[8] Chou CL Chang SC Lin TC Chen WS Jiang JK Wang HS. Differences in clinicopathological characteristics of colorectal cancer between younger and elderly patients: an analysis of 322 patients from a single institution. Am J Surg (2011) 202(5):574–82. doi: 10.1016/j.amjsurg.2010.10.014 21872205

[9] Glynne-Jones R Wyrwicz L Tiret E Brown G Rödel C Cervantes A. Rectal cancer: ESMO Clinical Practice Guidelines for diagnosis, treatment and follow-up. Ann Oncol (2017) 28(suppl_4):iv22–40. doi: 10.1093/annonc/mdx224 28881920

[10] Beets GL Figueiredo NF Beets-Tan RG. Management of rectal cancer without radical resection. Annu Rev Med (2017) 68:169–82. doi: 10.1146/annurev-med-062915-021419 27618750

[11] Garcia-Aguilar J Patil S Gollub MJ Kim JK Yuval JB Thompson HM. Organ preservation in patients with rectal adenocarcinoma treated with total neoadjuvant therapy. J Clin Oncol (2022) 40(23):2546–56. doi: 10.1200/JCO.22.00032 PMC9362876 35483010

[12] Roeder F Meldolesi E Gerum S Valentini V Rödel C. Recent advances in (chemo-)radiation therapy for rectal cancer: a comprehensive review. Radiat Oncol (2020) 15(1):262. doi: 10.1186/s13014-020-01695-0 33172475 PMC7656724

[13] Ferrari L Fichera A. Neoadjuvant chemoradiation therapy and pathological complete response in rectal cancer. Gastroenterol Rep (Oxf) (2015) 3(4):277–88. doi: 10.1093/gastro/gov039 PMC4650974 26290512

[14] Park IJ You YN Agarwal A Skibber JM Rodriguez-Bigas MA Eng C. Neoadjuvant treatment response as an early response indicator for patients with rectal cancer. J Clin Oncol (2012) 30(15):1770–6. doi: 10.1200/JCO.2011.39.7901 PMC3383178 22493423

[15] Glimelius B. Neo-adjuvant radiotherapy in rectal cancer. World J Gastroenterol (2013) 19(46):8489–501. doi: 10.3748/wjg.v19.i46.8489 PMC3870494 24379566

[16] Janjan NA Khoo VS Abbruzzese J Pazdur R Dubrow R Cleary KR. Tumor downstaging and sphincter preservation with preoperative chemoradiation in locally advanced rectal cancer: the M. D. Anderson Cancer Center experience. Int J Radiat Oncol Biol Phys (1999) 44(5):1027–38. doi: 10.1016/s0360-3016(99)00099-1 10421535

[17] Buckley AM Lynam-Lennon N O’Neill H O’Sullivan J. Targeting hallmarks of cancer to enhance radiosensitivity in gastrointestinal cancers. Nat Rev Gastroenterol Hepatol (2020) 17(5):298–313. doi: 10.1038/s41575-019-0247-2 32005946

[18] Bol V Bol A Bouzin C Labar D Lee JA Janssens G. Reprogramming of tumor metabolism by targeting mitochondria improves tumor response to irradiation. Acta Oncol (2015) 54(2):266–74. doi: 10.3109/0284186X.2014.932006 25007226

[19] Avantaggiati ML. Cancer metabolism as a therapeutic target: finding the right target(s) in the context of tumor heterogeneity, evolution, and metabolic plasticity. Oncol (Williston Park) (2013) 27(5):474. PMC4172372 25184272

[20] Tang L Wei F Wu Y He Y Shi L Xiong F. Role of metabolism in cancer cell radioresistance and radiosensitization methods. J Exp Clin Cancer Res (2018) 37(1):87. doi: 10.1186/s13046-018-0758-7 29688867 PMC5914062

[21] Buckley AM Dunne MR Lynam-Lennon N Kennedy SA Cannon A Reynolds AL. Pyrazinib (P3), [(E)-2-(2-Pyrazin-2-yl-vinyl)-phenol], a small molecule pyrazine compound enhances radiosensitivity in oesophageal adenocarcinoma. Cancer Lett (2019) 447:115–29. doi: 10.1016/j.canlet.2019.01.009 30664962

[22] Lynam-Lennon N Maher SG Maguire A Phelan J Muldoon C Reynolds JV. Altered mitochondrial function and energy metabolism is associated with a radioresistant phenotype in oesophageal adenocarcinoma. PloS One (2014) 9(6):e100738. doi: 10.1371/journal.pone.0100738 24968221 PMC4072695

[23] Buckley CE Yin X Meltzer S Ree AH Redalen KR Brennan L. Energy metabolism is altered in radioresistant rectal cancer. Int J Mol Sci (2023) 24(8):7082. doi: 10.3390/ijms24087082 37108244 PMC10138551

[24] Yu X Mao W Zhai Y Tong C Liu M Ma L. Anti-tumor activity of metformin: from metabolic and epigenetic perspectives. Oncotarget (2017) 8(3):5619–28. doi: 10.18632/oncotarget.13639 PMC5354934 27902459

[25] Foretz M Guigas B Bertrand L Pollak M Viollet B. Metformin: from mechanisms of action to therapies. Cell Metab (2014) 20(6):953–66. doi: 10.1016/j.cmet.2014.09.018 25456737

[26] Fontaine E. Metformin-induced mitochondrial complex I inhibition: facts, uncertainties, and consequences. Front Endocrinol (Lausanne) (2018) 9:753. doi: 10.3389/fendo.2018.00753 30619086 PMC6304344

[27] Skinner HD Crane CH Garrett CR Eng C Chang GJ Skibber JM. Metformin use and improved response to therapy in rectal cancer. Cancer Med (2013) 2(1):99–107. doi: 10.1002/cam4.54 24133632 PMC3797563

[28] Skinner HD McCurdy MR Echeverria AE Lin SH Welsh JW O'Reilly MS. Metformin use and improved response to therapy in esophageal adenocarcinoma. Acta Oncol (2013) 52(5):1002–9. doi: 10.3109/0284186X.2012.718096 22950385

[29] Van De Voorde L Janssen L Larue R Houben R Buijsen J Sosef M. Can metformin improve 'the tomorrow' of patients treated for oesophageal cancer? Eur J Surg Oncol (2015) 41(10):1333–9. doi: 10.1016/j.ejso.2015.05.012 26091848

[30] Jeong YK Kim MS Lee JY Kim EH Ha H. Metformin Radiosensitizes p53-Deficient Colorectal Cancer Cells through Induction of G2/M Arrest and Inhibition of DNA Repair Proteins. PloS One (2015) 10(11):e0143596. doi: 10.1371/journal.pone.0143596 26599019 PMC4657889

[31] Zannella VE Dal Pra A Muaddi H McKee TD Stapleton S Sykes J. Reprogramming metabolism with metformin improves tumor oxygenation and radiotherapy response. Clin Cancer Res (2013) 19(24):6741–50. doi: 10.1158/1078-0432.CCR-13-1787 24141625

[32] Franken NA Rodermond HM Stap J Haveman J van Bree C. Clonogenic assay of cells in *vitro* . Nat Protoc (2006) 1(5):2315–9. doi: 10.1038/nprot.2006.339 17406473

[33] Park J-H Kim Y-H Park E-H Lee S-J Kim H Kim A. Effects of metformin and phenformin on apoptosis and epithelial-mesenchymal transition in chemoresistant rectal cancer. Cancer Sci (2019) 110(9):2834–45. doi: 10.1111/cas.14124 PMC6726705 31278880

[34] Sinclair WK Morton RA. X-ray sensitivity during the cell generation cycle of cultured chinese hamster cells. Rad Res (1966) 29(3):450–74. doi: 10.2307/3572025 5924188

[35] Bhatt AN Chauhan A Khanna S Rai Y Singh S Soni R. Transient elevation of glycolysis confers radio-resistance by facilitating DNA repair in cells. BMC Cancer (2015) 15:335. doi: 10.1186/s12885-015-1368-9 25925410 PMC4425929

[36] Colen CB Seraji-Bozorgzad N Marples B Galloway MP Sloan AE Mathupala SP. Metabolic remodeling of malignant gliomas for enhanced sensitization during radiotherapy: an *in vitro* study. Neurosurgery (2006) 59(6):1313–23. doi: 10.1227/01.NEU.0000249218.65332.BF PMC3385862 17277695

[37] Leung E Cairns RA Chaudary N Vellanki RN Kalliomaki T Moriyama EH. Metabolic targeting of HIF-dependent glycolysis reduces lactate, increases oxygen consumption and enhances response to high-dose single-fraction radiotherapy in hypoxic solid tumors. BMC Cancer (2017) 17(1):418. doi: 10.1186/s12885-017-3402-6 28619042 PMC5473006

[38] Madhok BM Yeluri S Perry SL Hughes TA Jayne DG. Dichloroacetate induces apoptosis and cell-cycle arrest in colorectal cancer cells. Br J Cancer (2010) 102(12):1746–52. doi: 10.1038/sj.bjc.6605701 PMC2883702 20485289

[39] Yang Y Su D Zhao L Zhang D Xu J Wan J. Different effects of LDH-A inhibition by oxamate in non-small cell lung cancer cells. Oncotarget (2014) 5(23):11886–96. doi: 10.18632/oncotarget.2620 PMC4323009 25361010

[40] Hunter AJ Hendrikse AS Renan MJ. Can radiation-induced apoptosis be modulated by inhibitors of energy metabolism? Int J Radiat Biol (2007) 83(2):105–14. doi: 10.1080/09553000601121157 17357432

[41] Miller TW Soto-Pantoja DR Schwartz AL Sipes JM DeGraff WG Ridnour LA. CD47 receptor globally regulates metabolic pathways that control resistance to ionizing radiation. J Biol Chem (2015) 290(41):24858–74. doi: 10.1074/jbc.M115.665752 PMC4598996 26311851

[42] McCann E O'Sullivan J Marcone S. Targeting cancer-cell mitochondria and metabolism to improve radiotherapy response. Transl Oncol (2021) 14(1):100905. doi: 10.1016/j.tranon.2020.100905 33069104 PMC7562988

[43] Rao M Gao C Guo M Law BYK Xu Y. Effects of metformin treatment on radiotherapy efficacy in patients with cancer and diabetes: a systematic review and meta-analysis. Cancer Manage Res (2018) 10:4881–90. doi: 10.2147/CMAR.S174535 PMC6205529 30425579

[44] Samsuri NAB Leech M Marignol L. Metformin and improved treatment outcomes in radiation therapy: A review. Cancer Treat Rev (2017) 55:150–62. doi: 10.1016/j.ctrv.2017.03.005 28399491

[45] Lin A Maity A. Molecular pathways: A novel approach to targeting hypoxia and improving radiotherapy efficacy via reduction in oxygen demand. Clin Cancer Res (2015) 21(9):1995–2000. doi: 10.1158/1078-0432.CCR-14-0858 25934887 PMC4418024

[46] Mortezaee K Shabeeb D Musa AE Najafi M Farhood B. Metformin as a radiation modifier; implications to normal tissue protection and tumor sensitization. Curr Clin Pharmacol (2019) 14(1):41–53. doi: 10.2174/1574884713666181025141559 30360725

[47] Cheng G Zielonka J Ouari O Lopez M McAllister D Boyle K. Mitochondria-targeted analogues of metformin exhibit enhanced antiproliferative and radiosensitizing effects in pancreatic cancer cells. Cancer Res (2016) 76(13):3904. doi: 10.1158/0008-5472.CAN-15-2534 27216187 PMC4930686

[48] Kendziorra E Ahlborn K Spitzner M Rave-Fränk M Emons G Gaedcke J. Silencing of the Wnt transcription factor TCF4 sensitizes colorectal cancer cells to (chemo-) radiotherapy. Carcinogenesis (2011) 32(12):1824–31. doi: 10.1093/carcin/bgr222 PMC3254167 21983179

[49] Koerdel K Spitzner M Meyer T Engels N Krause F Gaedcke J. NOTCH Activation via gp130/STAT3 Signaling Confers Resistance to Chemoradiotherapy. Cancers (2021) 13(3):455. doi: 10.3390/cancers13030455 33530306 PMC7865718

[50] Loubiere C Clavel S Gilleron J Harisseh R Fauconnier J Ben-Sahra I. The energy disruptor metformin targets mitochondrial integrity via modification of calcium flux in cancer cells. Sci Rep (2017) 7(1):5040. doi: 10.1038/s41598-017-05052-2 28698627 PMC5506014

[51] Sivalingam VN Latif A Kitson S McVey R Finegan KG Marshall K. Hypoxia and hyperglycaemia determine why some endometrial tumours fail to respond to metformin. Br J Can (2020) 122(1):62–71. doi: 10.1038/s41416-019-0627-y PMC6964676 31819173

[52] Warkad MS Kim C-H Kang B-G Park S-H Jung J-S Feng J-H. Metformin-induced ROS upregulation as amplified by apigenin causes profound anticancer activity while sparing normal cells. Sci Rep (2021) 11(1):14002. doi: 10.1038/s41598-021-93270-0 34234193 PMC8263563

[53] Brown SL Kolozsvary A Isrow DM Al Feghali K Lapanowski K Jenrow KA. A novel mechanism of high dose radiation sensitization by metformin. Front Oncol (2019) 9:247. doi: 10.3389/fonc.2019.00247 31024849 PMC6465931

[54] Mogavero A Maiorana MV Zanutto S Varinelli L Bozzi F Belfiore A. Metformin transiently inhibits colorectal cancer cell proliferation as a result of either AMPK activation or increased ROS production. Sci Rep (2017) 7(1):15992. doi: 10.1038/s41598-017-16149-z 29167573 PMC5700100

[55] de Mey S Jiang H Corbet C Wang H Dufait I Law K. Antidiabetic biguanides radiosensitize hypoxic colorectal cancer cells through a decrease in oxygen consumption. Front Pharmacol (2018) 9:1073. doi: 10.3389/fphar.2018.01073 30337872 PMC6178882

[56] Fernandes JM Jandrey EHF Koyama FC Leite KRM Camargo AA Costa ÉT. Metformin as an alternative radiosensitizing agent to 5-fluorouracil during neoadjuvant treatment for rectal cancer. Dis Colon Rectum (2020) 63(7):918–26. doi: 10.1097/dcr.0000000000001626 32229782

[57] Kalyanaraman B Cheng G Hardy M Ouari O Sikora A Zielonka J. Mitochondria-targeted metformins: anti-tumour and redox signalling mechanisms. Interface Focus (2017) 7(2):20160109–. doi: 10.1098/rsfs.2016.0109 PMC5311906 28382202

[58] Buckley AM Dunne MR Morrissey ME Kennedy SA Nolan A Davern M. Real-time metabolic profiling of oesophageal tumours reveals an altered metabolic phenotype to different oxygen tensions and to treatment with Pyrazinib. Sci Rep (2020) 10(1):12105. doi: 10.1038/s41598-020-68777-7 32694701 PMC7374542

[59] Lan J Cadassou O Corbet C Riant O Feron O. Discovery of mitochondrial complex I inhibitors as anticancer and radiosensitizer drugs based on compensatory stimulation of lactate release. Cancers (2022) 14(21):5454. doi: 10.3390/cancers14215454 36358872 PMC9658316

[60] Strunz AM Peschke P Waldeck W Ehemann V Kissel M Debus J. Preferential radiosensitization in p53-mutated human tumour cell lines by pentoxifylline-mediated disruption of the G2/M checkpoint control. Int J Radiat Biol (2002) 78(8):721–32. doi: 10.1080/09553000210141667 12194756

[61] Anastasov N Höfig I Vasconcellos IG Rappl K Braselmann H Ludyga N. Radiation resistance due to high expression of miR-21 and G2/M checkpoint arrest in breast cancer cells. Radiat Oncol (2012) 7(1):206. doi: 10.1186/1748-717X-7-206 23216894 PMC3573984

[62] Khader E Ismail WW Mhaidat NM Alqudah MA. Effect of metformin on irinotecan-induced cell cycle arrest in colorectal cancer cell lines HCT116 and SW480. Int J Health Sci (2021) 15(5):34–41. PMC8434841 34548861

[63] Liu C Liu Q Yan A Chang H Ding Y Tao J. Metformin revert insulin-induced oxaliplatin resistance by activating mitochondrial apoptosis pathway in human colon cancer HCT116 cells. Cancer Med (2020) 9(11):3875–84. doi: 10.1002/cam4.3029 PMC7286444 32248666

[64] Marinello PC da Silva TN Panis C Neves AF Machado KL Borges FH. Mechanism of metformin action in MCF-7 and MDA-MB-231 human breast cancer cells involves oxidative stress generation, DNA damage, and transforming growth factor β1 induction. Tumour Biol (2016) 37(4):5337–46. doi: 10.1007/s13277-015-4395-x 26561471

[65] Sena P Mancini S Benincasa M Mariani F Palumbo C Roncucci L. Metformin induces apoptosis and alters cellular responses to oxidative stress in ht29 colon cancer cells: preliminary findings. Int J Mol Sci (2018) 19(5):1478. doi: 10.3390/ijms19051478 29772687 PMC5983851

[66] Vial G Detaille D Guigas B. Role of mitochondria in the mechanism(s) of action of metformin. Front Endocrinol (Lausanne) (2019) 10:294. doi: 10.3389/fendo.2019.00294 31133988 PMC6514102

[67] Wang LW Li ZS Zou DW Jin ZD Gao J Xu GM. Metformin induces apoptosis of pancreatic cancer cells. World J Gastroenterol (2008) 14(47):7192–8. doi: 10.3748/wjg.14.7192 PMC4988356 19084933

[68] He J Wang K Zheng N Qiu Y Xie G Su M. Metformin suppressed the proliferation of LoVo cells and induced a time-dependent metabolic and transcriptional alteration. Sci Rep (2015) 5:17423–. doi: 10.1038/srep17423 PMC4663508 26616174

[69] Liu C Nie J Wang R Mao W. The cell cycle G2/M block is an indicator of cellular radiosensitivity. Dose Response (2019) 17(4):1559325819891008. doi: 10.1177/1559325819891008 31839758 PMC6902394

[70] Pawlik TM Keyomarsi K. Role of cell cycle in mediating sensitivity to radiotherapy. Int J Radiat Oncol Biol Phys (2004) 59(4):928–42. doi: 10.1016/j.ijrobp.2004.03.005 15234026

[71] Chiang CF Chao TT Su YF Hsu CC Chien CY Chiu KC. Metformin-treated cancer cells modulate macrophage polarization through AMPK-NF-kappaB signaling. Oncotarget (2017) 8(13):20706–18. doi: 10.18632/oncotarget.14982 PMC5400538 28157701

[72] Cha JH Yang WH Xia W Wei Y Chan LC Lim SO. Metformin promotes antitumor immunity via endoplasmic-reticulum-associated degradation of PD-L1. Mol Cell (2018) 71(4):606–20.e7. doi: 10.1016/j.molcel.2018.07.030 30118680 PMC6786495

[73] Tsukioki T Shien T Tanaka T Suzuki Y Kajihara Y Hatono M. Influences of preoperative metformin on immunological factors in early breast cancer. Cancer Chemother Pharmacol (2020) 86(1):55–63. doi: 10.1007/s00280-020-04092-2 32533334 PMC7338817

[74] Galon J Mlecnik B Bindea G Angell HK Berger A Lagorce C. Towards the introduction of the 'Immunoscore' in the classification of malignant tumours. J Pathol (2014) 232(2):199–209. doi: 10.1002/path.4287 24122236 PMC4255306

[75] Neagu M Constantin C Popescu ID Zipeto D Tzanakakis G Nikitovic D. Inflammation and metabolism in cancer cell-mitochondria key player. Front Oncol (2019) 9:348. doi: 10.3389/fonc.2019.00348 31139559 PMC6527883

[76] Isvoranu G Surcel M Munteanu AN Bratu OG Ionita−Radu F Neagu MT. Therapeutic potential of interleukin−15 in cancer (Review). Exp Ther Med (2021) 22(1):675. doi: 10.3892/etm.2021.10107 33986840 PMC8112152

[77] Pilones K Aryankalayil J Formenti S Demaria S. Intratumoral IL-15 potentiates radiation-induced anti-tumor immunity. J Immunother Cancer (2015) 3(2):239. doi: 10.1186/2051-1426-3-S2-P239

[78] Tojo M Miyato H Koinuma K Horie H Tsukui H Kimura Y. Metformin combined with local irradiation provokes abscopal effects in a murine rectal cancer model. Sci Rep (2022) 12(1):7290. doi: 10.1038/s41598-022-11236-2 35508498 PMC9068771

