# Supplementary material for: Metformin is a metabolic modulator and radiosensitiser in rectal cancer
Source: Front Oncol. 2023 Aug 3;13:1216911. doi: 10.3389/fonc.2023.1216911 (PMC10435980; doi:10.3389/fonc.2023.1216911)
Supplement: Supplementary file 1 [file Table_1.docx]

Supplementary Material

Metformin is a metabolic modulator and radiosensitiser in rectal cancer

**Croí E. Buckley, Rebecca M. O’Brien, Timothy S. Nugent, Noel E. Donlon, Fiona O’Connell, John V. Reynolds, Adnan Hafeez, Diarmuid S. O’Ríordáin, Robert A. Hannon, Paul Neary, Reza Kalbassi, Brian J. Mehigan, Paul H. McCormick, Cara Dunne, Michael E. Kelly, John O. Larkin, Jacintha O’Sullivan and Niamh Lynam-Lennon^*^**

*** Correspondence:** Corresponding Author: Niamh Lynam-Lennon [lynamlen@tcd.ie](mailto:lynamlen@tcd.ie)

**Supplementary Table S1: Canonical pathways significantly altered in metformin-treated SW837 cells.**

| **Ingenuity Canonical Pathways** | **-log(*p*-value)** | **Molecules** |
| --- | --- | --- |
| Sirtuin Signaling Pathway | 5.08 | ACSS2,ATG101,ATG14,ATP5PF,H1-4,MT-ND5,NDUFA2,NDUFA6,NDUFB1,NDUFB10,NDUFS2,SMARCA5,TIMM8A,TIMM8B,TOMM34,TUBA1B,TUBA1C,TUBA4A |
| Oxidative Phosphorylation | 4.49 | ATP5MG,ATP5PF,COX5A,CYCS,MT-ND5,NDUFA2,NDUFA6,NDUFB1,NDUFB10,NDUFS2 |
| Mitochondrial Dysfunction | 4.14 | ATP5MG,ATP5PF,COX5A,CYCS,GLRX2,MT-ND5,NDUFA2,NDUFA6,NDUFB1,NDUFB10,NDUFS2,RHOT2 |
| Huntington's Disease Signaling | 4.08 | ATF4,ATP5PF,CASP2,CYCS,GNB1L,GNG12,POLR2G,PRKD3,PSMB7,PSMC5,PSMD13,PSMD4,PSME2,RASA1,REST,YKT6 |
| Remodeling of Epithelial Adherens Junctions | 3.62 | CLIP1,CTNNA1,CTNND1,TUBA1B,TUBA1C,TUBA4A,TUBB2B |
| Germ Cell-Sertoli Cell Junction Signaling | 3.49 | CTNNA1,CTNND1,ITGA6,PLS1,RHOT2,SORBS1,TJP1,TUBA1B,TUBA1C,TUBA4A,TUBB2B |
| Hypoxia Signaling in the Cardiovascular System | 3.31 | ATF4,BIRC6,HSP90AA1,HSP90B1,P4HB,UBE2E1,VEGFA |
| FAT10 Signaling Pathway | 3.29 | NUB1,PSMB7,PSMC5,PSMD13,PSMD4,PSME2 |
| Estrogen Receptor Signaling | 3.19 | ATF4,FOS,GNB1L,GNG12,HSP90AA1,HSP90B1,IGF2R,MED24,MT-ND5,NCOA1,NDUFA2,NDUFA6,NDUFB1,NDUFB10,NDUFS2,PRKD3,TRRAP,VEGFA |
| Unfolded protein response | 2.84 | ATF4,DNAJB14,DNAJC10,EIF2A,HSP90B1,INSIG1,P4HB |
| Sertoli Cell-Sertoli Cell Junction Signaling | 2.79 | CTNNA1,ITGA6,ITGB5,PLS1,SORBS1,SPTBN1,TJP1,TUBA1B,TUBA1C,TUBA4A,TUBB2B |
| Protein Ubiquitination Pathway | 2.71 | BIRC6,DNAJB14,DNAJC10,ELOB,HSP90AA1,HSP90B1,PSMB7,PSMC5,PSMD13,PSMD4,PSME2,UBE2E1,USP39 |
| NER (Nucleotide Excision Repair, Enhanced Pathway) | 2.5 | H4C3,H4C8,POLA2,POLD2,POLE3,POLR2G,RFC4 |
| Arsenate Detoxification I (Glutaredoxin) | 2.47 | GLRX2,GSTO1 |
| BAG2 Signaling Pathway | 2.37 | HSP90AA1,PSMB7,PSMC5,PSMD13,PSMD4,PSME2 |
| GDP-mannose Biosynthesis | 2.3 | GPI,PMM2 |
| Coronavirus Replication Pathway | 2.1 | TUBA1B,TUBA1C,TUBA4A,TUBB2B |
| Sphingomyelin Metabolism | 2.04 | SGMS1,SMPD4 |
| Ferroptosis Signaling Pathway | 1.98 | ATF4,EIF2A,H2AX,PEBP1,RB1,SLC38A1,TXNRD1 |
| Ephrin Receptor Signaling | 1.84 | ATF4,GNB1L,GNG12,ITGA6,ITGB5,KALRN,RASA1,SORBS1,VEGFA |
| Superpathway of Cholesterol Biosynthesis | 1.82 | HMGCS1,MSMO1,SQLE |
| Xenobiotic Metabolism AHR Signaling Pathway | 1.78 | ALDH1L2,GSTO1,HSP90AA1,HSP90B1,NCOA1 |
| Role of BRCA1 in DNA Damage Response | 1.75 | PBRM1,PLK1,RB1,RFC4,UIMC1 |
| Transcriptional Regulatory Network in Embryonic Stem Cells | 1.73 | H4C3,H4C8,HNF4A,REST |
| NRF2-mediated Oxidative Stress Response | 1.72 | ATF4,DNAJB14,DNAJC10,FOS,GSTO1,HSP90AA1,HSP90B1,PRKD3,TXNRD1 |
| EIF2 Signaling | 1.71 | ATF4,EIF2A,EIF4G1,EIF4G3,EIF5,MT-RNR2,RPL41,TRIB3,VEGFA |
| Autophagy | 1.7 | ATF4,ATG101,ATG14,BIRC6,EIF2A,FOS,GNB1L,RB1CC1,VEGFA |
| Endocannabinoid Cancer Inhibition Pathway | 1.68 | ATF4,CASP2,EIF2A,GNB1L,SMPD4,TRIB3,VEGFA |
| Cholesterol Biosynthesis I | 1.62 | MSMO1,SQLE |
| Cholesterol Biosynthesis II (via 24,25-dihydrolanosterol) | 1.62 | MSMO1,SQLE |
| Cholesterol Biosynthesis III (via Desmosterol) | 1.62 | MSMO1,SQLE |
| HER-2 Signaling in Breast Cancer | 1.59 | COX5A,FOS,GNB1L,ITGB5,NDC1,PRKD3,RAE1,RB1,TPR |
| Chondroitin Sulfate Degradation (Metazoa) | 1.56 | GALNS,OGA |
| Colanic Acid Building Blocks Biosynthesis | 1.56 | GPI,PMM2 |
| Oxytocin In Brain Signaling Pathway | 1.52 | ATF4,EIF2A,GNB1L,GNG12,ITPR2,PLAAT3,PNPLA8,PRKD3 |
| Mitotic Roles of Polo-Like Kinase | 1.51 | HSP90AA1,HSP90B1,PLK1,SMC3 |
| Gap Junction Signaling | 1.51 | CSNK1G3,ITPR2,PRKD3,TJP1,TUBA1B,TUBA1C,TUBA4A,TUBB2B |
| ILK Signaling | 1.5 | ATF4,DOCK1,DSP,FOS,ITGB5,MYO18A,RHOT2,VEGFA |
| 14-3-3-mediated Signaling | 1.49 | FOS,PRKD3,TUBA1B,TUBA1C,TUBA4A,TUBB2B |
| Epoxysqualene Biosynthesis | 1.43 | SQLE |
| CXCR4 Signaling | 1.42 | DOCK1,FOS,GNB1L,GNG12,ITPR2,PRKD3,RHOT2 |
| Molecular Mechanisms of Cancer | 1.4 | CDK13,CTNNA1,CTNND1,CYCS,DVL1,FOS,GNB1L,GNG12,ITGA6,ITGB5,PRKD3,RASA1,RB1,RHOT2 |
| HIF1α Signaling | 1.4 | ELOB,GPI,HSP90AA1,NAA10,NCOA1,PRKD3,SLC2A8,VEGFA |
| Androgen Signaling | 1.39 | GNB1L,GNG12,HSP90AA1,ITPR2,NCOA1,POLR2G,PRKD3 |
| Axonal Guidance Signaling | 1.34 | DOCK1,GNB1L,GNG12,ITGA6,ITGB5,KALRN,KLC1,NFAT5,PRKD3,RASA1,TUBA1B,TUBA1C,TUBA4A,TUBB2B,VEGFA |
| PPAR Signaling | 1.33 | FOS,HSP90AA1,HSP90B1,IL18,NCOA1 |
| Hereditary Breast Cancer Signaling | 1.32 | H2AX,PALB2,PBRM1,POLR2G,RB1,RFC4 |
| α-Adrenergic Signaling | 1.3 | GNB1L,GNG12,GYS1,ITPR2,PRKD3 |

Biostatistical analysis was performed on significantly altered genes in metformin treated SW837 cells, when compared to H_2_O vehicle control by IPA analysis to identify predicted altered canonical pathways. Statistical analysis was performed by right-tailed Fisher’s exact test using IPA analysis.

**Supplementary Table S2:** Effect of metformin treatment (10 mM) on secreted inflammatory mediators in conditioned media from non-cancer rectal tissue and rectal cancer tissue, when compared to vehicle control.

|  |  | **Non-Cancer (*n* =12)** | **Cancer (*n* =12)** |
| --- | --- | --- | --- |
|  | **Mediator** | ***p*-value** | ***p*-value** |
| Angiogenesis Panel | FGF(basic) | 0.27 | 0.73 |
|  | Flt-1 | 0.85 | 0.38 |
|  | PIGF | 0.08 | 0.97 |
|  | Tie-2 | 0.75 | >0.99 |
|  | VEGF | 0.97 | 0.30 |
|  | VEGF-C | 0.58 | 0.90 |
|  | VEGF-D | N/A | >0.99 |
| Chemokine Panel | Eotaxin | 0.44 | 0.15 |
|  | Eotaxin-3 | N/A | 0.25 |
|  | IP-10 | 0.43 | >0.99 |
|  | MCP-1 | 0.62 | 0.47 |
|  | MCP-4 | 0.13 | 0.62 |
|  | MDC | >0.99 | 0.34 |
|  | MIP-1α | 0.81 | *0.04 |
|  | MIP-1β | 0.43 | 0.34 |
|  | TARC | 0.23 | >0.99 |
| Cytokine Panel 1 | GM-CSF | 0.15 | 0.27 |
|  | IL-1α | 0.30 | *0.02 |
|  | IL-5 | 0.08 | *0.01 |
|  | IL-7 | 0.75 | 0.16 |
|  | IL-23p40 | 0.47 | 0.20 |
|  | IL-15 | 0.65 | *0.04 |
|  | IL-16 | 0.73 | *0.04 |
|  | IL-17A | *0.03 | 0.37 |
|  | TNF-β | N/A | 0.25 |
| Cytokine Panel 2 | IL-17A/F | 0.25 | 0.38 |
|  | IL-17B | *0.03 | *0.04 |
|  | IL-17C | N/A | N/A |
|  | IL-17D | *0.04 | 0.79 |
|  | IL-1RA | 0.79 | 0.20 |
|  | IL-3 | 0.38 | 0.25 |
|  | IL-9 | N/A | N/A |
|  | TSLP | 0.56 | 0.62 |
| TH-17 Panel | IL-21 | 0.50 | N/A |
|  | IL-22 | 0.79 | 0.18 |
|  | IL-23 | 0.43 | 0.08 |
|  | IL-27 | >0.99 | N/A |
|  | IL-31 | 0.31 | N/A |
|  | MIP-3α | 0.15 | 0.73 |
| Pro-Inflammatory Panel | IFN-γ | 0.52 | 0.85 |
|  | IL-1β | 0.91 | 0.11 |
|  | IL-2 | 0.62 | 0.13 |
|  | IL-4 | 0.11 | 0.42 |
|  | IL-6 | 0.05 | 0.47 |
|  | IL-8 | 0.27 | 0.09 |
|  | IL-10 | 0.68 | 0.73 |
|  | IL-12p70 | 0.20 | 0.52 |
|  | IL-13 | 0.38 | 0.09 |
|  | TNF-α | 0.47 | 0.15 |
| Vascular Injury Panel | CRP | 0.85 | *0.02 |
|  | sICAM-1 | 0.62 | 0.79 |
|  | sVCAM-1 | 0.57 | 0.20 |
|  | SAA | 0.85 | 0.05 |

*p*-values reflect differences in levels of inflammatory mediators in Metformin treated versus vehicle control samples; N/A, Statistical analysis could not be performed as inflammatory mediator was detected in less than *n*=3 samples per group; *p< 0.05.
